# The Overlooked Stereoisomers of the Ionizable Lipid ALC315

**DOI:** 10.1021/jacs.5c08345

**Published:** 2025-08-01

**Authors:** Chandra Kanta De, Masumi Tsuda, Chendan Zhu, Stefanie Dehn, Heike Hinrichs, Nobuya Tsuji, Hui Jin, Hisashi Arase, Shinya Tanaka, Benjamin List

**Affiliations:** † Max-Planck-Institut für Kohlenforschung, Kaiser-Wilhelm-Platz 1, D−45470, Mülheim an der Ruhr, Germany; ‡ Department of Cancer Pathology, Faculty of Medicine, Hokkaido University, N15 W7, Kita-ku, Sapporo, 060-8638, Japan; § Institute for Chemical Reaction Design and Discovery (ICReDD), Hokkaido University, N21 W10, Kita-ku, Sapporo, 001-0021, Japan; ∥ Department of Immunochemistry, Research Institute for Microbial Diseases, 13013 Osaka University, Suita, Osaka 565-0871, Japan; ⊥ Laboratory of Immunochemistry, WPI Immunology Frontier Research Center, 13013 Osaka University, Suita, Osaka 565-0871, Japan; # Center for Advanced Modalities and DDS, 13013 Osaka University, Suita, Osaka 565-0871, Japan; 7 Center for Infectious Disease Education and Research, 13013 Osaka University, Suita, Osaka 565-0871, Japan

## Abstract

Lipid nanoparticles (LNPs) are a powerful delivery platform for nucleic acid therapeutics such as mRNA vaccines and gene therapies. Central to their success are ionizable lipids, which facilitate the cellular uptake and endosomal escape of nucleic acids. However, achieving a high delivery efficiency often comes with the drawback of increased cytotoxicity. Here, we report a chemical, biological, and toxicological investigation into the three stereoisomers of ALC315, a mixture of which constitutes one of the most successful marketed ionizable lipids for LNPs. We demonstrate that the individual stereoisomers of ALC315 can be accessed by either the asymmetric chemical synthesis or chromatography of an intermediate. An LNP formulation based on a single stereoisomer of ALC315 enhances mRNA transfer efficiency while reducing the associated cytotoxicity in human cell lines. Our results underscore the potential of stereochemically pure ionizable lipids as key components in the development of next-generation nucleic acid therapies, offering an enhanced delivery performance and better safety profiles.

## Graphical abstract

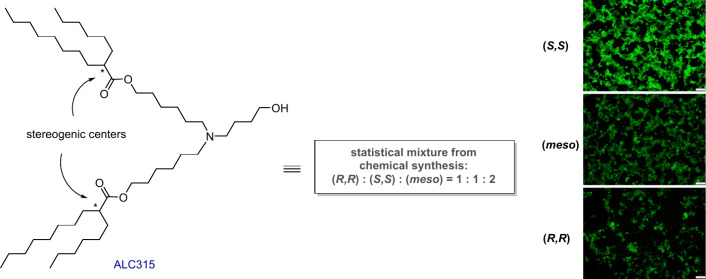

Lipid nanoparticles (LNPs) have emerged as a versatile and efficient platform for delivering nucleic acids, particularly in the context of mRNA vaccines and gene therapies.^1–6^ The COVID-19 pandemic demonstrated the immense potential of LNPs for rapid vaccine deployment, with ionizable lipids playing a critical role in mediating cellular uptake, nucleic acid release, and minimizing cytotoxicity.^7,8^ Among the various ionizable lipids used in current LNP formulations, ALC315 ^9^ (((4-hydroxybutyl)­azanediyl)­bis­(hexane-6,1-diyl)­bis­(2-hexyldecanoate), Figure 1) has garnered significant attention due to its favorable balance between efficacy and safety. However, while ALC315 has been successfully employed in numerous applications, there remains room for improvement in terms of delivery efficiency and selectivity, stability, and the overall therapeutic index.^10–15^ For example, modifying ALC315 to enhance packing behavior in LNPs or cell selectivity offers a promising strategy to improve the delivery of nucleic acids. As a result of its symmetry and two stereogenic centers, ALC315 consists of a mixture of three stereoisomers, the enantiomers (*R,R*)-ALC315 and (*S,S*)-ALC315 and an achiral meso-form, (*meso*)-ALC315 or (*R,S*)-ALC315. To the best of our knowledge, stereochemically pure ALC315 isomers have not previously been described. Consequently, their individual chemical and biological properties are unknown. Given that over 1.2 billion doses of the Pfizer-BioNTech COVID-19 vaccine (Comirnaty) have been administered worldwide and the fact that stereoisomeric substances are well-known to be distinguishable by all live forms, we became interested in the question of how the ALC315 stereochemistry affects the overall efficacy and safety of the corresponding LNP therapeutic applications.

**Figure 1:**
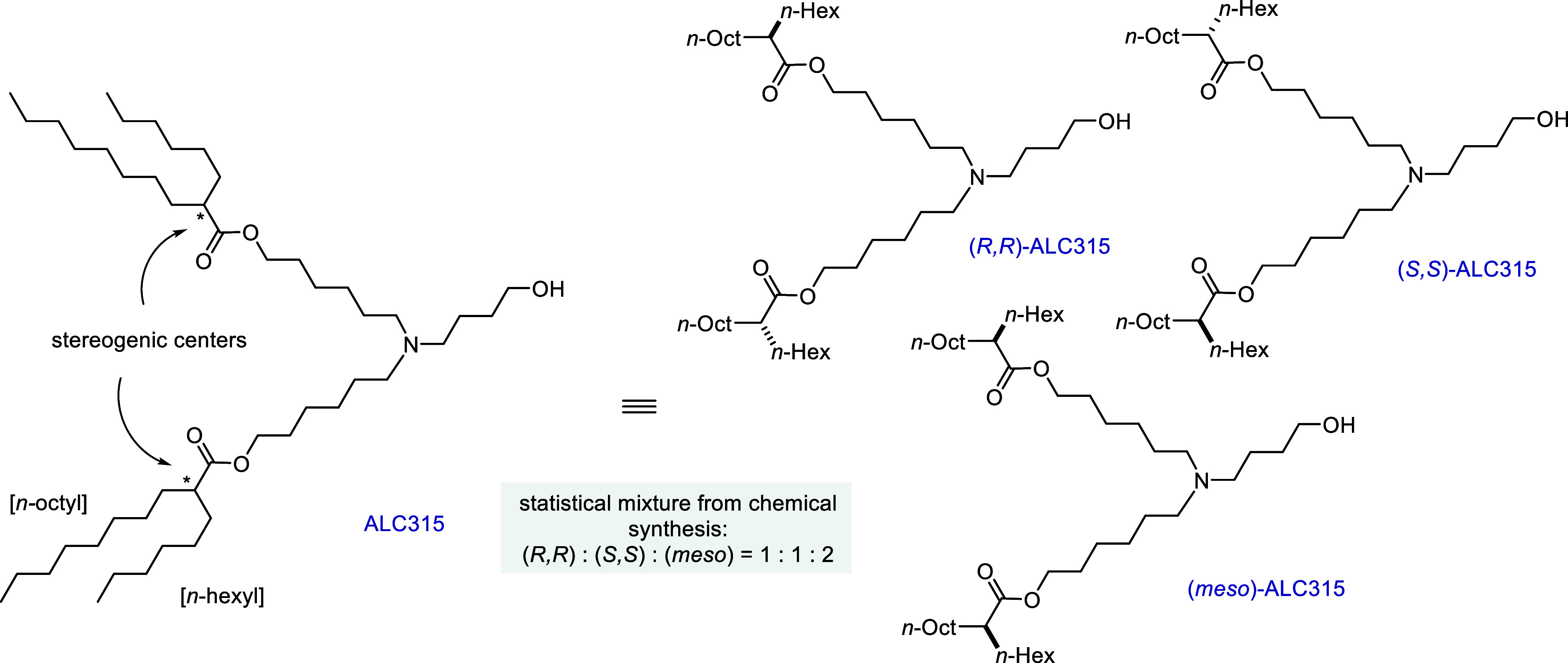
Stereoisomers of ALC315. ALC315 has two identical stereogenic centers and as a result exists as three different stereoisomers, (*R*,*R*)-ALC315, (*S*,*S*)-ALC315, and (*meso*)-ALC315.

This report details the asymmetric chemical synthesis of the three stereoisomers of ALC315, involving a diastereoselective alkylation or preparative chromatographic stereoisomer separation of the corresponding enantiopure acids. We also describe in vitro and in vivo evaluations that reveal different cytotoxicities of the LNPs derived from these stereoisomers and provide a safer platform for future therapeutic and vaccine developments. These findings have broad implications for the advancement of LNP technologies.

The subtle and perhaps overlooked chirality of ALC315 rests upon the two stereogenic centers in the alpha positions of each of its two ester functional groups (Figure 1). Each of these two centers features a H atom, an *n*-octyl chain, and an *n*-hexyl chain. The chiral branch, in turn, is a result not of design, but of the availability of the alpha-branched carboxylic acids, which are used in the chemical synthesis of ALC315. Technically, such branched carboxylic acids are produced by dimerizing two identical aldehydes in an aldol condensation, followed by hydrogenation and oxidation,^16^ or in a Guerbet reaction of two identical alcohols, followed by oxidation.^17^ In either case, the characteristic chain length difference of two methylene units and the resulting chirality of these technically produced branched carboxylic acids arise as a consequence of their chemical synthesis.

At the onset of our investigations, it was unclear whether the stereoisomers of ALC315, which have not previously been reported or mentioned, could even be separated or synthetically accessed. We reasoned that the Myers alkylation of pseudoephedrine-derived amides would be a promising approach, as it has previously been used for the asymmetric synthesis of alpha-branched carboxylic acid derivatives, featuring two unactivated *n*-alkyl groups.^18,19^ Indeed, when (1*S*,2*S*)-pseudoephedrine was reacted with *n*-octyl carboxylic acid chloride, the corresponding amide derivative was obtained in high yield. This intermediate was then treated with LDA under cryogenic conditions, and the resulting enolate was alkylated with *n*-octyl iodide followed by an acidic hydrolysis delivering the enantioenriched product (*R*)-**1** (98:2 er) on a multigram scale. A similar reaction sequence was performed to obtain enantioenriched carboxylic acid (*S*)-**1**. First, (1*S*,2*S*)-pseudoephedrine was treated with *n*-decyl carboxylic acid chloride followed by alkylation using *n*-hexyl iodide, and finally, an acidic hydrolysis gave the enantioenriched product (*S*)-**1** (98.5:1.5 er). Alternatively, the enantiopure carboxylic acids could also be obtained by preparative HPLC separation using a chiral stationary phase (see the SI). Having both enantioenriched carboxylic acids **1** in hand, we next aimed to complete the synthesis of the three stereoisomerically pure ALC315 lipid variants. However, the reported technical route^9^ for the synthesis of ALC315 cannot be used toward (*meso*)-ALC315, and introduction of the valuable carboxylic acid in the early phase of the synthesis is not ideal. Hence, we have designed a new synthetic route that addresses these issues. We have identified compound **5** as an ideal building block for the synthesis of all three stereoisomerically pure lipids. Protected amino alcohol **3** was treated with hydroxyaldehyde **4** under reductive amination conditions, delivering the desired compound **5** in high yield. Steglich esterification between compound **5** and enantioenriched acid (*R*)-**1** followed by removal of the TBS protecting group under acidic conditions gave (*R,R*)-ALC315. A similar reaction sequence was performed with enantioenriched (*S*)-**1** and furnished (*S,S*)-ALC315. To obtain (*meso*)-ALC315, two sequential Steglich esterifications were performed with the two enantiopure acids. First, compound **5** was treated with (*R*)-**1** to obtain monoester **6**, which was isolated and then subjected to another Steglich esterification with (*S*)-**1**, followed by deprotection (Figure 2). With the three stereochemically pure ALC315 variants in hand, we next assembled the corresponding LNPs either following the known injection method or using the NanoAssembler Ignite system (see the SI).

**Figure 2:**
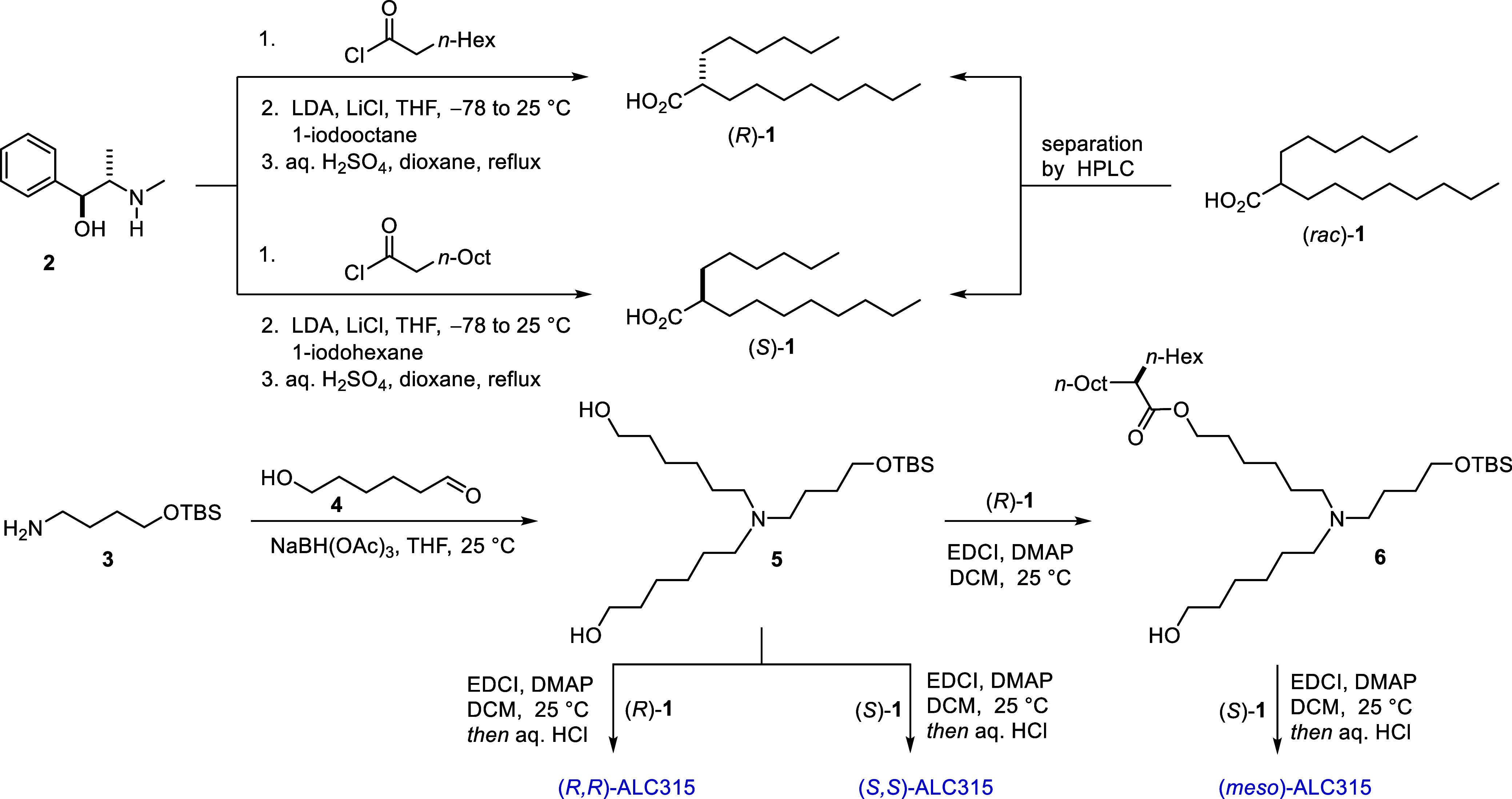
Asymmetric chemical synthesis of the three stereoisomers of ALC315.

To investigate the transfection efficiency of the ALC315 stereoisomers, LNPs were generated including mRNA of the green fluorescence protein (GFP) and transfected into human embryonal kidney cells 293T (HEK293T), and the expression levels of GFP were analyzed by fluorescence-activated cell sorting (FACS) after 16 h of transfection. For all three ALC315 isomers, GFP was successfully expressed in HEK293T cells, and no significant differences were observed (Figure 3a). At the same time, cell viability was analyzed by uptake of propidium iodide (PI) in cells incubated with LNPs. Surprisingly, it was found that there were some unviable cell populations in cells incubated with ALC315 stereoisomers (Figure 3b). Accordingly, we were interested in investigating the cytotoxicity of the isomers at different concentrations.

**Figure 3:**
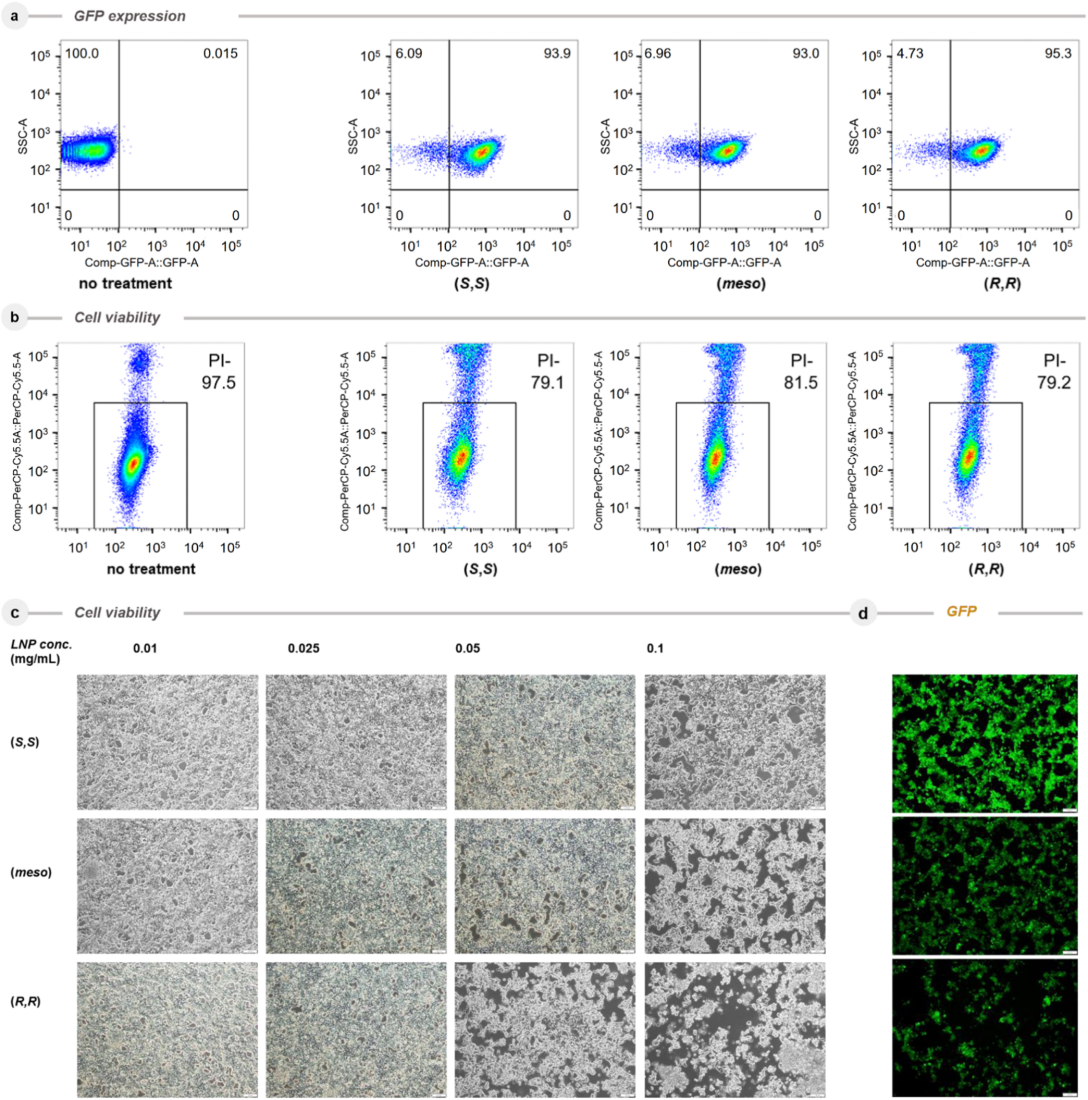
Transfection of the GFP expression plasmid into human embryonic kidney 293T cells with LNPs from stereochemically pure ALC315 isomers. **a,** HEK293T cells were treated with (*S,S*)-, (*meso*)-, and (*R,R*)-ALC315 LNPs for 16 h, and expressions of GFP were measured by FACS and indicated on the horizontal axis. Vertical axis indicates side scatter (SSC) for cell size. **b**, Cell viabilities were indicated by incorporation of propidium iodide (PI) as indicated on the vertical bar. Horizontal axis indicates side scatter (SSC) analysis for the cell size. Arrows indicate inviable cell clusters that have taken up PI. **c**, Cell viability of the HEK293T cell line. **d**, Expressions of GFP of the HEK293T cell line.

The effects of different concentrations of 0.010, 0.025, 0.050, and 0.100 mg/mL of the three LNP variants on HEK293T cells for 48 h were examined and summarized in Figure 3c. Notably, it was found that at a low concentration of LNPs (0.01–0.05 mg/mL) cell viability was not affected for the (*S,S*)- and (*meso*)-isomers with full confluence. However, in the case of the (*R,R*)-isomer, cells were significantly affected. Further investigation at higher concentration (0.10 mg/mL) of LNPs showed that the cell density reduced to approximately 60% with the (*R,R*)-isomer, while cells seemed to be moderately affected with the (meso)-isomer and with the (*S,S*)-isomer only a slight reduction of cell viability was observed.

The GFP fluorescence was clearly visualized in the case of the (*S,S*)-isomer, but less detectable in both the (*meso*)- and (*R,R*)-isomers in accordance with the remaining cell numbers (Figure 3d). Even at low cell numbers in the case of both the (*meso*)- and (*R,R*)-isomer, the protein expressions of GFP were detectable at 0.01–0.10 mg/mL with a certain difference based on the amount of transfected LNPs.

Continuing our investigation of the cytotoxicity of LNPs based on the ALC315 stereoisomers, cell viability experiments were performed using 10 different human cancer cell lines (SI Table 1). All the cell lines were individually incubated with LNPs based on ALC315 isomers at a concentration of 0.05 and 0.10 mg/mL for 48 h, and the cell viabilities were measured (Figure 4a and 4b). Indeed, different cell viabilities were detected for different isomers of ALC315. For example, cytotoxicity of ALC315 isomers on the KMG4 cell line at a concentration of 0.05 mg/mL of LNP showed that the (*S*,*S*)-isomer had virtually no effect on cell viability and the (*meso*)-isomer was slightly toxic with 97.7% cell viability, while the (*R*,*R*)-isomer provided significantly lower cell viability (84.4%). In contrast, at 0.10 mg/mL concentration, the (*S*,*S*)- and (*meso*)-isomers were found to be toxic, with cell viabilities of 77.3% and 75.6%, respectively. However, for the (*R*,*R*)-isomer, the cell viability was reduced more to only 63.5%.

**Figure 4:**
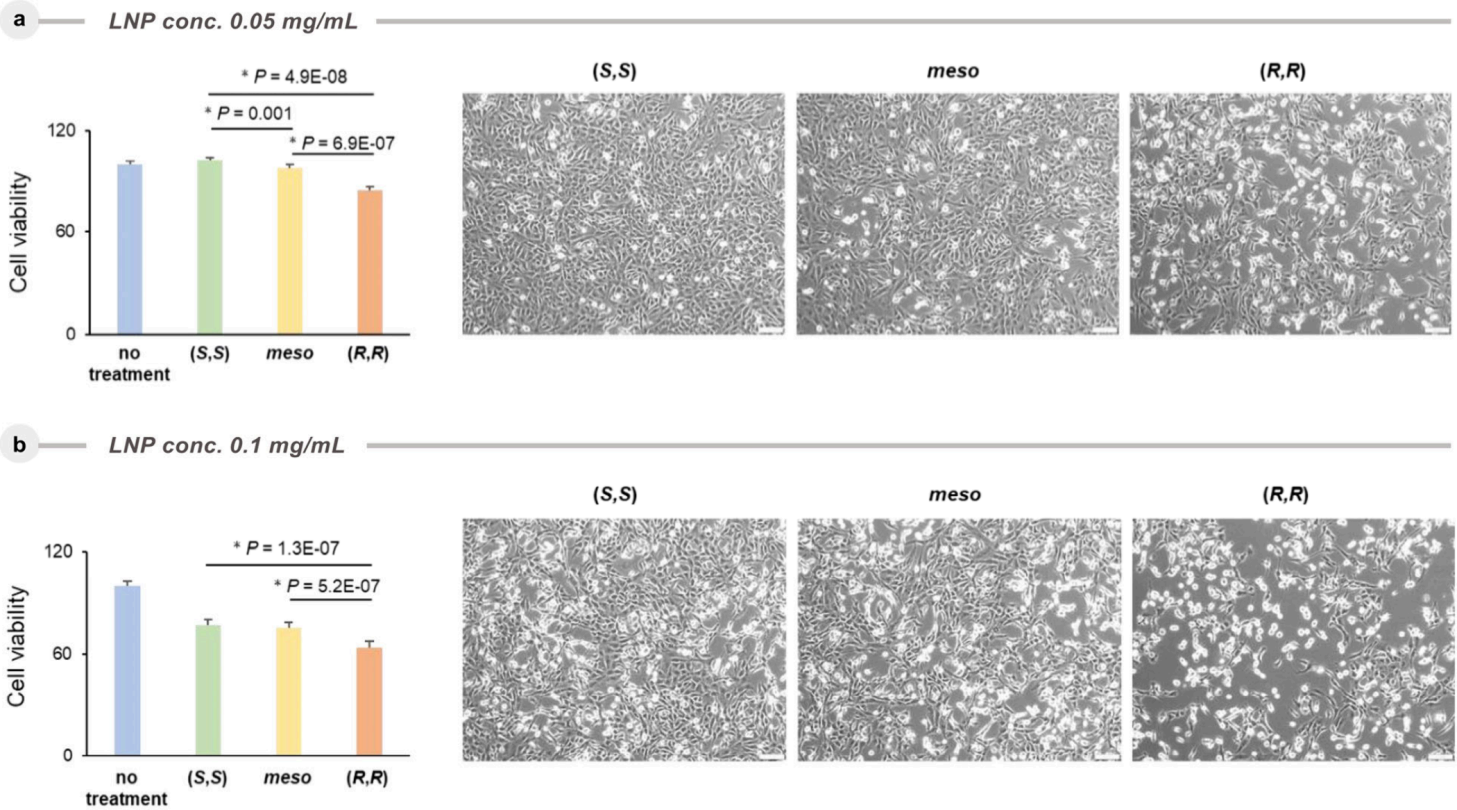
Cell viabilities of the KMG4 cell line treated with LNPs from stereoisomers of ALC315: (*S,S*), (*meso*), and (*R,R*). **a**, Cell viability of the KMG4 cell line at 0.05 mg/mL concentration. **b,** Cell viability of the KMG4 cell line at 0.10 mg/mL concentration.

It is worth mentioning that a longer incubation time (72 h) further increases the toxicity of the (*R*,*R*)-isomer. A similar toxicity profile pattern was also observed for other cell lines (SI Figure 3). Among these 10 human cancer cell lines, six cell lines, namely, KMG4, A549, HCA7, Fuji, U87, and J82, showed that ALC315-induced cytotoxicity was mild for the (*S*,*S*)-isomer, as 80% cell viabilities were detected even at a higher concentration of LNPs (0.10 mg/mL). However, for four other cell lines, namely, SYO-1, HeLa, HepG2, and Jurkat, cell viabilities were equally affected with all three ALC315 isomers (Sl Figure 3).

Since the (*R*,*R*)-isomer of ALC315 was found to be more toxic than the corresponding (*meso*)- and (*S*,*S*)-isomers to cells, we have analyzed the expression levels of representative inflammation-related genes, such as *IL-6*, *TNF-α*, and *INF*-γ-*R1* in Jurkat cells that are well responsible for cytokines. Interestingly, it was found that after incubation with 0.10 mg/mL LNPs for 22 h, the mRNA expression levels of these genes were indeed higher in the case of the (*R*,*R*)-isomer than those with the two other isomers (Figure 5b).^20–22^

**Figure 5:**
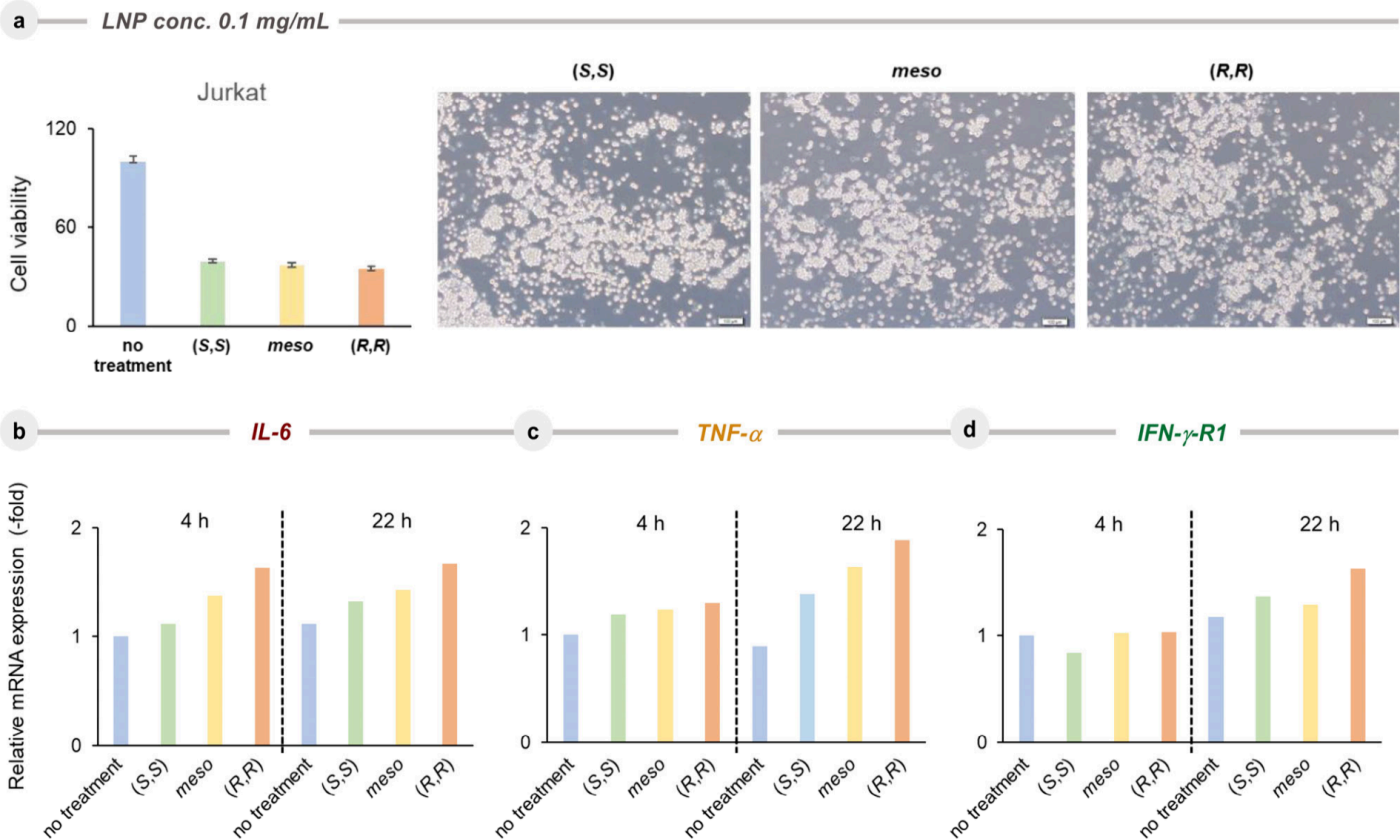
Expression of inflammation-related genes in Jurkat cells treated with (*S,S*)-, (*meso*)-, and (*R,R*)-ALC315 by RT-PCR. Jurkat cells were treated with ALC isomers at a concentration of 0.10 mg/mL. **a,** Cell viabilities after 48 h were displayed as the upper bar graph and photographs. **b,** mRNA expression levels of *IL-6*, **(c)** *TNF-α*, and **(d)** *IFN*-γ-*R1* were analyzed after 4 and 22 h.

We identified the stereochemistry of ALC315 as an opportunity to improve LNP formulations for safer and more efficient nucleic acid delivery. Our joined synthetic and biological investigation reveals that of the three stereoisomers, the (*S*,*S*)-enantiomer features the lowest cytotoxicity and, consequently, the highest mRNA transfer efficiency. In fact, the (*R*)-stereochemistry appears to be associated with increased cytotoxicity, following the trend (*S,S*)- < (*meso*)- < (*R,R*)-ALC315, as observed in experiments conducted in biological duplicates. To date, no studies have systematically examined the biological effects of lipid stereoisomers, precluding definitive mechanistic conclusions. However, as phosphatidylserine externalization is a key regulator of apoptosis,^23^ differential interactions with this lipid may contribute to cell damage. Moreover, recent reports indicate that cysteine stereoisomers in lipid nanoparticles can modulate cytotoxicity via caspase activation^24^ or through stereoselective engagement with cellular receptors such as the LDL receptor. While the mechanism behind this phenomenon is currently unclear and is the topic of ongoing investigations in our laboratories, the recommendation should be to only use the safer and more efficient (*S*,*S*)-ALC315 ionizable lipid in future drug deliveries.^25^

## Supplementary Material



## Notes

Experimental and mechanistic details and analytical data for all new compounds (PDF)
