# Supplementary material for: The Overlooked Stereoisomers of the Ionizable Lipid ALC315
Source: J Am Chem Soc. 2025 Aug 1;147(32):28595–600. doi: 10.1021/jacs.5c08345 (PMC12356529; doi:10.1021/jacs.5c08345)
Supplement: Supplementary file 1 [file ja5c08345_si_001.pdf]

# Supplementary Material

## The Overlooked Stereoisomers of the Ionizable Lipid ALC315

Chandra Kanta De†[1], Masumi Tsuda†[2,3], Chendan Zhu†[1], Stefanie Dehn[1], Heike Hinrichs[1], Nobuya Tsuji[3], Hui Jin[4], Hisashi Arase[4,5,6,7], Shinya Tanaka[2,3]*, Benjamin List*

[1]Max-Planck-Institut für Kohlenforschung, Kaiser-Wilhelm-Platz 1, D–45470 Mülheim an der Ruhr, Germany.

[2] Department of Cancer Pathology, Faculty of Medicine, Hokkaido University, N15 W7, Kita-ku, Sapporo, 060–8638, Japan.

[3]Institute for Chemical Reaction Design and Discovery (ICReDD), Hokkaido University, N21 W10, Kita-ku, Sapporo, 001–0021, Japan.

[4]Department of Immunochemistry, Research Institute for Microbial Diseases, Osaka University, Suita, Osaka 565–0871, Japan.

[5]Laboratory of Immunochemistry, WPI Immunology Frontier Research Center, Osaka University, Suita , Osaka 565–0871, Japan.

[6]Center for Advanced Modalities and DDS, Osaka University, Suita, Osaka 565–0871, Japan

[7]Center for Infectious Disease Education and Research, Osaka University, Suita, Osaka 565–0871, Japan

# Contents


1. General Information S2

2. Synthesis of (*R*)-2-hexyldecanoic acid: S4

3. Synthesis of (*S*)-2-hexyldecanoic acid S6

4. Synthesis of 6,6'-((4-((tert-butyldimethylsilyl)oxy)butyl)azanediyl)bis(hexan-1-ol) (**5**) S7

5. Synthesis of (*R*,*R*)-ALC315 S8

6. Synthesis of (*S*,*S*)-ALC315 S9

7. Synthesis of (*meso*)-ALC315 S10

8. Methods and Methods for Biology S11

9. NMR Spectra S15

10. HPLC Traces S26

11. References S28

# 1. Supplementary Methods

## General Information

Unless otherwise stated, all reagents were purchased from commercial suppliers and used without further purification. All solvents used in the reactions were distilled from appropriate drying agents prior to use. Reactions were monitored by thin layer chromatography (TLC) on silica gel pre-coated plastic sheets (0.2 mm, Macherey-Nagel). Visualization was accomplished by irradiation with UV light at 254 nm and/or *p*-anisaldehyde, CAM and PMA stain. Column chromatography was performed on Merck silica gel (60, particle size 0.040–0.063 mm). $^{1}H$ and $^{13}C$ NMR spectra were recorded on a Bruker AV-500 spectrometer in deuterated solvents. Proton chemical shifts are reported in ppm (δ) relative to the solvent resonance employed as the internal standard ($CDCl_3$ and $CD_2Cl_2$ δ 7.26 and 5.32 ppm, respectively). Data are reported as follows: chemical shift, multiplicity (s = singlet, d = doublet, t = triplet, q = quartet, p = pentet, s = sextet, h = heptet, m = multiplet, br = broad), coupling constants (Hz) and integration. $^{13}C$ chemical shifts are reported in ppm with the solvent resonance as the internal standard ($CDCl_3$ and $CD_2Cl_2$ δ 77.16 and 54.00 ppm respectively). High resolution mass spectra were determined on a Bruker APEX III FTMS (7 T magnet). Optical rotations were determined with an Autopol IV polarimeter (Rudolph Research Analytical) at 589 nm and 25 °C. Data are reported as follows: $[\alpha]_{\lambda}^{temp}$, concentration (*c* in g/100 mL), and solvent. Enantiomeric ratios (er) were determined by HPLC analysis employing a chiral stationary phase column specified in the individual experiment, by comparing the samples with the appropriate racemic mixtures.

## 2. Synthesis of (*R*)-2-hexyldecanoic acid:

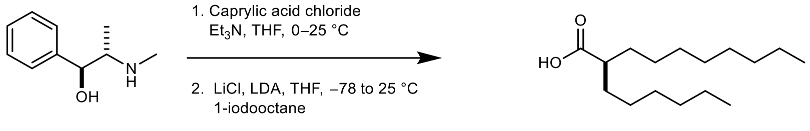


**2** **(*R*)-1**

***Step 1:*** N-((*1S,2S*)-1-hydroxy-1-phenylpropan-2-yl)-N-methyloctanamide (**7**):

A flame dried 250 mL two-neck flask was charged with (+)-pseudoephedrine (4.0 g, 24.2 mmol, 1.0 equiv.) and

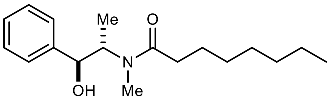


**7**

triethylamine (4.8 mL, 27.8 mmol, 1.3 equiv.). To this flask, anhydrous tetrahydrofuran (50 mL) was added and the reaction mixture was cooled to 0 °C. After 10 min at 0 °C, an ice-cold solution of caprylic acid chloride (4.8 mL, 27.8 mmol, 1.15 equiv.) in tetrahydrofuran (10 mL) was added via addition funnel over 10 min. Then the reaction mixture was further stirred for 30 min and water (10 mL) was added to quench the excess acid chloride. The reaction mixture was then diluted with brine (80 mL) and extracted with ethyl acetate (3 x 100 mL). The combined organic layers was extracted with brine (2 × 80 mL), dried over $Na_2SO_4$ and concentrated under reduced pressure. The crude retraction mixture was purified by flash column chromatography on silica. The title compound was obtained as a colorless oil (6.0 g, 85% yield).

**$^1$H NMR** (501 MHz, $CDCl_3$, 3:1 amide rotamers were observed) δ 7.41–7.24 (m, 5H), 4.58 (dd, $J$ = 14.3, 8.2 Hz, 1H), 4.50–4.29 (m, 1H), 2.93–2.77 (3H, with 2.91 (s, 0.74H) and 2.80 (s, 2.24H)), 2.47–2.19 (m, 2H), 1.70–1.53 (m, 2H), 1.36–1.21 (m, 8H), 1.16–0.95 (2H, with 1.12 (d, $J$ = 6.9 Hz, 2.24H) and 0.98 (d, $J$ = 6.8 Hz, 0.74H)), 0.92–0.84 (m, 3H).

**$^{13}$C NMR** (126 MHz, $CDCl_3$) δ 175.9, 174.6, 142.7, 141.3, 128.9, 128.6, 128.5, 127.7, 127.1, 126.5, 76.8, 75.7, 59.1, 58.5, 34.6, 33.9, 33.3, 31.9, 31.9, 29.7, 29.5, 29.3, 29.2, 26.8, 25.5, 25.2, 22.8, 15.5, 14.6, 14.3, 14.2.

**HRMS** *m/z* (ESI): calcd. for $C_{18}H_{30}NO_2$ $[M+H]^+$: 292.2271; found: 292.2270.

***Step 2:*** (*R*)-2-hexyl-N-((*1S,2S*)-1-hydroxy-1-phenylpropan-2-yl)-N-methyldecanamide (**8**):

A flame dried 250 mL two-neck flask was charged with a magnetic stirrer and anhydrous lithium chloride (3.2 g, 76.1

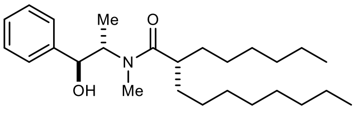


**8**

mmol, 6.0 equiv.). To the reaction flask, anhydrous tetrahydrofuran (15 mL) and diisopropylamine (4.0 mL, 28.6 mmol, 2.25 equiv.) were added and the suspension was cooled to –78 °C. After 10 min, a solution of *n*-butyllithium in hexanes (2.8 M, 9.4 mL, 26.4 mmol, 2.08 equiv.) was added slowly through the cold side wall of the flask. The reaction mixture was warmed to 0 °C for 10 min and then again was cooled to –78 °C. After 10 min, an ice-cold solution of N-((*1S,2S*)-1-hydroxy-1-phenylpropan-2-yl)-N-methyloctanamide (3.7 g, 12.7 mmol, 1.0 equiv.) in tetrahydrofuran (55 mL) was added slowly. The reaction mixture was further stirred at –78 °C for 1 h, at 0 °C for 15 min, and at rt for 5 min. After that1-iodooctane (17.7 mL, 149 mmol, 1.50 equiv.) was added at 0 °C and stirring was continued for additional 15 min at 0 °C. The reaction mixture was then quenched by the addition of saturated aq. $NH_4Cl$ solution at 0 °C. The reaction mixture was transferred to a separation funnel and was diluted with saturated aq.

$NH_4Cl$ solution (80 mL). The aqueous layer was extracted with ethyl acetate (3 x 100 mL) and the combined organic layers were dried over $Na_2SO_4$ and concentrated. The crude product was purified by flash column chromatography on silica (eluent: 5% EtOAc to 15% EtOAc in hexanes *v/v*). The title compound was obtained as an oil (2.9 g, 89% yield).

**$^1$H NMR** (501 MHz, $CDCl_3$, 5.6:1 amide rotamers were observed ) δ 7.44–7.22 (m, 5H), 4.60 (dd, *J* = 23.9, 8.1 Hz, 1H), 4.49–4.09 (m, 1H), 2.95–2.80 (3H, with 2.92 (s, 0.45H) and 2.84 (s, 2.55H)), ), 2.80–2.47 (1 H, with 2.80–2.73 (m, 0.15H) and 2.53 (tt, *J* = 8.7, 5.4 Hz, 0.85H)), 1.82–0.94 (m, 30H), 0.91–0.82 (m, 6H).

**$^{13}$C NMR** (126 MHz, $CDCl_3$) δ 179.1, 177.7, 142.8, 141.3, 128.9, 128.6, 128.4, 127.6, 127.1, 126.4, 76.6, 75.6, 60.3, 58.3, 42.5, 41.6, 33.2, 33.2, 32.9, 32.0, 32.0, 31.9, 30.1, 30.0, 29.7, 29.6, 29.5, 29.4, 28.0, 27.8, 27.6, 27.1, 22.8, 22.8, 22.7, 15.6, 14.7, 14.2, 14.2 (other signals not detected or observed).

**HRMS** *m/z* (ESI): calcd. for $C_{26}H_{45}NO_2Na$ $[M+Na]^+$: 426.3342; found: 426.3343.

***Step 3:*** (*R*)-2-hexyldecanoic acid:

A 50 mL one-neck flask was charged with a magnetic stirrer and (*R*)-2-hexyl-N-((*1S,2S*)-1-hydroxy-1-phenylpropan-2-yl)-N-methyldecanamide (1.2 g, 3.0 mmol, 1.0 equiv.). The compound was dissolved in 1,4-dioxane (6 mL) and aq. $H_2SO_4$ acid (18 M, 6 mL) was added slowly. After that it was heated at 110 °C for 1 h. The reaction mixture was cooled to 0 °C and carefully basified to pH >10 using aq. sodium hydroxide (50%, (w/w)) solution and then further diluted with water (10 mL). The aqueous layer was extracted with dichloromethane (3 x 10 mL). The aqueous layer was then acidified to pH < 2 using aq. $H_2SO_4$ acid (6 M) solution. Finally, the aqueous layer was extracted with dichloromethane (3 × 20 mL), was dried over $Na_2SO_4$ and was concentrated under reduced pressure. The crude product was purified by flash column chromatography on silica (eluent: 20% ethyl acetate in hexanes *v/v*). (*R*)-2-hexyldecanoic acid was obtained as a colorless oil (0.57 g, 75% yield).

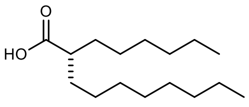


(*R*)-1

**$^1$H NMR** (501 MHz, $CDCl_3$) δ 11.12 (br s, 1H), 2.34 (tt, *J* = 8.7, 5.4 Hz, 1H), 1.70–1.55 (m, 2H), 1.54–1.41 (m, 2H), 1.37–1.19 (m, 20H), 0.91–0.84 (m, 6H).

**$^{13}$C NMR** (126 MHz, $CDCl_3$) δ 182.6, 45.6, 32.3, 32.0, 31.8, 29.7, 29.6, 29.4, 29.4, 27.5, 27.5, 22.8, 22.7, 14.2, 14.2.

**HRMS** *m/z* (ESI): calcd. for $C_{16}H_{31}O_2$ $[M–H]^–$: 255.2330; found: 255.2332.

The obtained enantioenriched acid was treated with (*R*)-1-Phenylethylamine under amide coupling conditions and the obtained amide derivative was used for HPLC analysis. The enantiomeric ratio was measured by HPLC, Chiralpak AD-3, heptane/isopropanol = 99:1 (v/v), flow rate = 1.0 mL/min, λ = 215 nm, 298 K, $t_R$ = 11.5 min (major) and $t_R$ = 13.0 min (minor). er = 98:2.

## 3. Synthesis of (*S*)-2-hexyldecanoic acid:

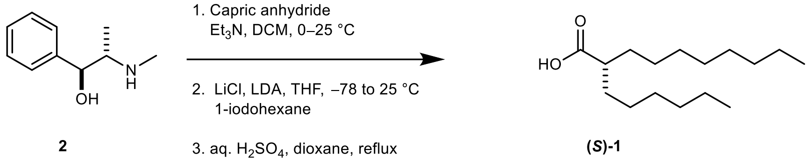


**2** (*S*)**-1**

***Step 1*****:** N-((*1S*,*2S*)-1-hydroxy-1-phenylpropan-2-yl)-N-methyldecanamide (**9**):

A flame dried 100 mL two-neck flask was charged with a magnetic stir bar and (+)-pseudoephedrine (2.0 g, 12.1 mmol, 1.0 equiv.). Anhydrous dichloromethane (24 mL) and triethylamine (2.1 mL, 14.5 mmol, 1.2 equiv.) were added at rt. The reaction flask was cooled to 0 °C and capric anhydride (4.8 mL, 13.0 mmol, 1.1 equiv.) was added over 10 min. After 3 h, water (1 mL) was added to quench the excess anhydride. The reaction mixture was then diluted with brine (40 mL) and was extracted with dichloromethane (3 × 20 mL). The combined organic layers were dried over $Na_2SO_4$ and concentrated under reduced pressure. The crude product was purified by flash column chromatography on silica. The title compound was obtained as a colorless oil (3.5 g, 95% yield).

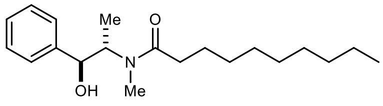


**9**

**$^1$H NMR** (501 MHz, $CDCl_3$, 2.8:1 amide rotamers were observed) δ 7.44–7.19 (m, 5H), 4.56 (dd, *J* = 13.6, 8.3 Hz, 1H), 4.48–3.92 (m, 1H), 2.92–2.75 (3H, with 2.89 (s, 0.8H) and 2.79 (s, 2.2H)), 2.45–2.16 (m, 2H), 1.70–1.50 (m, 2H), 1.40–1.18 (m, 12H), 1.14–0.93 (3H, with 1.10 (d, *J* = 7.1 Hz, 2.2H) and 0.97 (d, *J* = 6.8 Hz, 0.8H)), 0.87 (t, *J* = 6.9 Hz, 3H).

**$^{13}$C NMR** (126 MHz, $CDCl_3$) δ 175.8, 174.6, 142.6, 141.4, 128.8, 128.4, 127.7, 127.0, 126.5, 76.7, 75.6, 58.5, 34.5, 33.9, 32.0, 29.7, 29.6, 29.6, 29.6, 29.6, 29.5, 29.4, 29.4, 26.8, 25.5, 25.1, 22.8, 15.5, 14.6, 14.2.

**HRMS** *m/z* (ESI): calcd. for $C_{20}H_{33}NO_2Na$ $[M+Na]^+$: 342.2403; found: 342.2404.

***Step 2*****:** (*S*)-2-hexyl-N-((*1S*,*2S*)-1-hydroxy-1-phenylpropan-2-yl)-N-methyldecanamide (10):

The title compound was synthesized following the same procedure as mentioned above for (*R*)-acids step 2. The desired product was obtained as a colorless oil (85% yield).

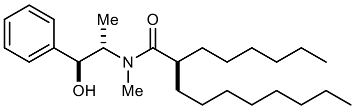


**10**

**$^1$H NMR** (501 MHz, $CD_2Cl_2$, 5.2:1 amide rotamers were observed) δ 7.43–7.17 (m, 5H), 4.66–4.52 (m, 1H), 4.34–4.04 (m, 1H), 2.90–2.77 (3H, with 2.87 (s, 0.48H) and 2.79 (s, 2.52H)) 2.77–2.47 (1H, with 2.77–2.71 (m, 0.15H) and 2.52 (tt, *J* = 8.5, 5.3 Hz, 0.85H)), 1.72–0.94 (m, 30H), 0.92–0.84 (m, 6H).

**$^{13}$C NMR** (126 MHz, $CD_2Cl_2$) δ 179.2, 143.7, 129.2, 128.7, 127.8, 127.6, 126.8, 79.2, 76.8, 58.6, 42.8, 33.6, 33.2, 32.5, 32.3, 30.4, 30.1, 29.9, 28.1, 28.0, 27.9, 23.2, 23.2, 15.8, 14.8, 14.4. (other signals not detected or observed).

**HRMS** *m/z* (ESI): calcd. for $C_{26}H_{46}NO_2$ $[M+H]^+$: 404.3523; found: 404.3523.

***Step 3*:** (*S*)-2-hexyldecanoic acid:

The title compound was synthesized following the same procedure as mentioned above for (*R*)-acids step 3. The desired product was obtained as a colorless oil (0.61 g, 80% yield).

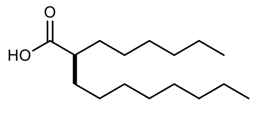

(S)-1

**$^1$H NMR** (501 MHz, $CD_2Cl_2$) δ 10.80 (br s, 1H), 2.34 (tt, *J* = 8.7, 5.4 Hz, 1H), 1.67–1.54 (m, 2H), 1.54–1.41 (m, 2H), 1.38–1.20 (m, 20H), 0.88 (t, *J* = 6.9 Hz, 6H).

**$^{13}$C NMR** (126 MHz, $CD_2Cl_2$) δ 182.4, 46.0, 32.8, 32.4, 32.3, 30.1, 30.0, 29.8, 29.8, 27.9, 27.9, 23.2, 23.2, 14.4, 14.4.

**HRMS** *m/z* (EI): calcd. for $C_{16}H_{32}O_2$ $[M]^+$: 256.2397; found: 256.2398.

The obtained enantioenriched acid was treated with (*R*)-1-Phenylethylamine under amide coupling conditions and the obtained amide derivative was used for HPLC analysis. The enantiomeric ratio was measured by HPLC, Chiralpak AD-3, heptane/isopropanol = 99:1 (v/v), flow rate = 1.0 mL/min, λ = 215 nm, 298 K, $t_R$ = 11.3 min (minor) and $t_R$ = 12.5 min (major). er = 98.5:1.5.

**Note: Preparative Chiral HPLC Separation:**

Racemic 2-hexyldecanoic acid (CAS No: 25354–97–6) was purched from Sigma-Aldrich and was used as received. We were interested to separate the enantiomers of the *rac*- 2-hexyldecanoic acid using chiral HPLC methods. However, extensive screening of the HPLC conditions under normal phase, did not provide any successful separation. Fortunately, under reverse phase HPLC condition using Chiralpak-IG-3R, acetonitrile/water (0.1% TFA) = 65:35 (v/v). flow rate = 1.0 mL/min, the enantiomers were separated.

## 4. Synthesis of 6,6'-((4-((tert-butyldimethylsilyl)oxy)butyl)azanediyl)bis(hexan-1-ol) (5):

A 250 mL one-neck flask was charged with magnetic stirrer and 4-((tert-butyldimethylsilyl)oxy)butan-1-amine (3.6 g, 17.6 mmol, 1.0 equiv.). To this reaction flask, THF (35 mL) and 6-hydroxyhexanal (4.5 g, 38.7 mmol, 2.5 equiv.) were added sequentially. The reaction mixture was stirred at rt for 10 min and then $NaHB(OAc)_3$ (11.2 g, 52.8 mmol, 4.0 equiv.) was added in small portions over 1 h at rt and stirring was continued for overnight at rt. The reaction mixture was cooled to 0 °C and was carefully neutralized with saturated aq. $NaHCO_3$ solution. The reaction mixture was extracted with ethyl acetate (3 x 100 mL), the combined organic layers were dried over $Na_2SO_4$ and was concentrated under reduced pressure. The crude reaction mixture was purified by flash column chromatography on silica (eluent: 100% acetone). Upon evaporation of the solvent under reduced pressure, the title compound was obtained as a light yellow oil (5.3 g, 78% yield).

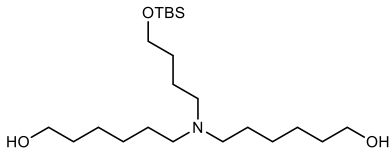


5

**$^1$H NMR** (501 MHz, $CD_2Cl_2$) δ 3.59 (dt, *J* = 13.2, 6.4 Hz, 6H), 2.36 (dt, *J* = 7.6, 6.0 Hz, 6H), 1.68 (br s, 2H), 1.56–1.27 (m, 20H), 0.89 (s, 9H), 0.04 (s, 6H).

**$^{13}$C NMR** (126 MHz, $CD_2Cl_2$) δ 63.5, 63.1, 54.4, 33.3, 31.3, 27.7, 27.5, 26.1, 26.1, 23.8, 18.6, –5.2.

**HRMS** *m/z* (ESI): calcd. for $C_{22}H_{50}NO_3Si$ $[M+H]^+$: 404.3554; found: 404.3556.

## 5. Synthesis of ((4-hydroxybutyl)azanediyl)bis(hexane-6,1-diyl) (*2R,2'R*)-bis(2-hexyldecanoate):

A flame dried 100 mL round bottom flask was charged with a magnetic stir bar, 6,6'-((4-((tert-butyldimethylsilyl)oxy)butyl)azanediyl)bis(hexan-1-ol) (1.0 g, 2.48 mmol, 1.0 equiv.) and anhydrous dichloromethane (30 mL). The reaction mixture was cooled to 0 °C and sequentially, (*R*)-2-hexyldecanoic acid (1.34 g, 5.2 mmol, 2.1 equiv., 98:2 er), DMAP (0.7 g, 5.5 mmol, 2.2 equiv.) and 1-ethyl-3-(3-dimethyllaminopropyl)carbodiimide hydrochloride (1.1 g, 5.5 mmol, 2.2 equiv.) were added. After 15 min at 0 °C, the reaction mixture was warmed up to rt and was further stirred overnight at rt. The reaction mixture was cooled to 0 °C and diluted with saturated aq. sodium bicarbonate solution (20 mL), brine (50 mL) and was extracted with dichloromethane (3 x 50 mL). The combined organics were dried over $Na_2SO_4$, filtered and concentrated under reduced pressure. The crude product was purified by flash column chromatography on silica (eluent: 20% ethyl acetate in hexanes to 40% ethyl acetate in hexanes *v*/*v*). The title compound was obtained as a colorless oil which was used for deprotection step without further purification.

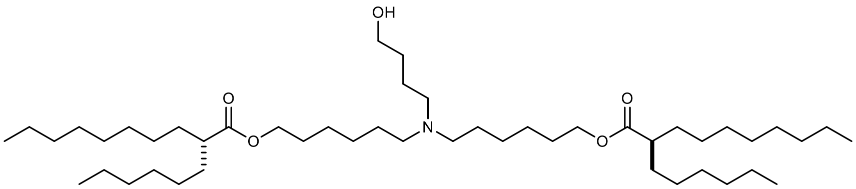

**(*R,R*)-ALC315**

((4-((tert-butyldimethylsilyl)oxy)butyl)azanediyl)bis(hexane-6,1-diyl) (*2R,2'R*)-bis(2-hexyldecanoate) was dissolved in tetrahydrofuran (15 mL) and was cooled to 0 °C. After 5 min at 0 °C, aq. HCl (10% *v/v*, 5 mL) was added and stirring was continued for additional 30 min. The mixture was carefully quenched with aq. Sat. $NaHCO_3$ to a basic pH and was extracted with dichloromethane (3 x 50 mL). The combined organic layers were dried over $Na_2SO_4$, filtered and concentrated under reduced pressure. The crude product was purified by flash column chromatography on silica (eluent: 50% ethyl acetate in hexanes to 100% ethyl acetate *v*/*v*). The title compound was obtained as a colorless oil (1.4 g, 74% yield over two steps).

**$^1$H NMR** (501 MHz, $CD_2Cl_2$-$d_2$) δ 4.03 (t, *J* = 6.6 Hz, 4H), 3.51–3.43 (m, 2H), 2.45–2.36 (m, 6H), 2.29 (tt, *J* = 9.0, 5.4 Hz, 2H), 1.65–1.22 (m, 69H), 0.88 (t, *J* = 6.9 Hz, 12H).

**$^{13}$C NMR** (126 MHz, $CD_2Cl_2$-$d_2$) δ 176.9, 64.5, 63.1, 55.2, 54.2, 46.4, 33.4, 33.1, 32.5, 32.3, 30.2, 30.0, 29.8, 29.8, 29.3, 28.0, 28.0, 27.8, 26.8, 26.6, 26.5, 23.3, 23.2, 14.5, 14.4.

**HRMS** *m/z* (ESI): calcd. for $C_{48}H_{96}NO_5$ $[M+H]^+$: 766.7283; found: 766.7284.

**Determination of enantiomeric ratio:**

The enantiomeric ratio was measured by HPLC after derivatizations of the lipid to carboxylic acid following the reaction sequence as below:

The obtained enantioenriched acid was treated with (*R*)-1-Phenylethylamine under amide coupling conditions and the obtained amide derivative was used for HPLC analysis.

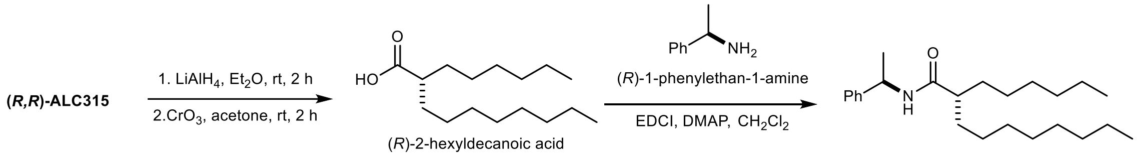


er ≥ 95.5:4.5 and calculated dr = >20:1.

## 6. Synthesis of ((4-hydroxybutyl)azanediyl)bis(hexane-6,1-diyl) (*2S,2'S*)-bis(2-hexyldecanoate):

Following the procedure for (*R*,*R*)-ALC315 and using (*S*)-2-hexyldecanoic acid (1.2 g, 3.0 mmol scale), the title compound was obtained as a colorless oil (1.8 g, 79% yield over two steps).

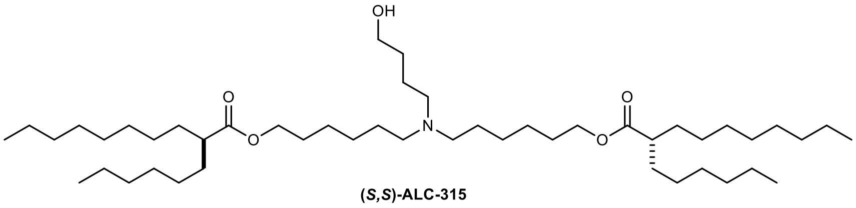


**$^1$H NMR** (501 MHz, $CD_2Cl_2$-*d*$_2$) δ 4.03 (t, *J* = 6.6 Hz, 4H), 3.48 (t, *J* = 5.0 Hz, 2H), 2.47–2.36 (m, 6H), 2.29 (tt, *J* = 8.8, 5.4 Hz, 2H), 1.65–1.21 (m, 69H), 0.88 (t, *J* = 6.9 Hz, 12H).

**$^{13}$C NMR** (126 MHz, $CD_2Cl_2$-*d*$_2$) δ 176.9, 64.5, 63.1, 55.2, 54.2, 46.4, 33.4, 33.1, 32.5, 32.3, 30.2, 30.0, 29.9, 29.8, 29.3, 28.0, 28.0, 27.8, 26.8, 26.6, 26.5, 23.3, 23.2, 14.5, 14.4.

**HRMS** *m/z* (ESI): calcd. for $C_{48}H_{96}NO_5$ $[M+H]^+$: 766.7283; found: 766.7285.

er ≥ 98:2 and calculated dr = >20:1.

## 7. Synthesis of 6-((6-(((*R*)-2-hexyldecanoyl)oxy)hexyl)(4-hydroxybutyl)amino)hexyl (*S*)-2-hexyldecanoate:

**6-((4-hydroxybutyl)(6-hydroxyhexyl)amino)hexyl (*R*)-2-hexyldecanoate (6):**

A flame dried 250 mL round bottom flask was charged with a magnetic stir bar, 6,6'-((4-((tert-butyldimethylsilyl)oxy)butyl)azanediyl)bis(hexan-1-ol) (2.0 g, 5.0 mmol, 1.3 equiv.) and anhydrous dichloromethane (50 mL). The reaction mixture was cooled to 0 °C and sequentially, (*R*)-2-hexyldecanoic acid (0.97 g, 3.8 mmol, 1.0 equiv., 98:2 er), DMAP (0.56 g, 4.5 mmol, 1.2 equiv.) and 1-ethyl-3-(3-dimethyllaminopropyl)carbodiimide hydrochloride (0.87 g, 4.5 mmol, 1.2 equiv.) were added. After 15 min at 0 °C, the reaction mixture was warmed up to rt and was further stirred overnight at rt. The reaction mixture was cooled to 0 °C and diluted with saturated aq. sodium bicarbonate solution (20 mL), brine (50 mL) and was extracted with dichloromethane (3 x 50 mL). The combined organics were dried over $Na_2SO_4$, filtered and concentrated under reduced pressure. The crude product was purified by flash column chromatography on silica (eluent: 50% diethyl ether in acetone *v*/*v*). The title compound was obtained as a colorless oil which was used for deprotection step without further purification.

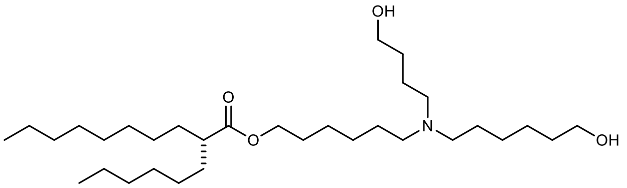

**6**

**$^1$H NMR** (501 MHz, $CD_2Cl_2$) δ 4.03 (t, *J* = 6.6 Hz, 2H), 3.59 (dt, *J* = 13.4, 6.5 Hz, 4H), 2.50–2.32 (m, 6H), 2.32–2.26 (m, 1H), 1.65–1.23 (m, 45H), 0.91–0.85 (m, 15H), 0.04 (s, 6H).

**$^{13}$C NMR** (126 MHz, $CD_2Cl_2$) δ 176.9, 64.6, 63.7, 63.3, 54.6, 54.6, 54.5, 46.4, 33.5, 33.1, 32.5, 32.3, 31.5, 30.2, 30.1, 29.9, 29.8, 29.4, 28.0, 28.0, 27.9, 27.8, 27.8, 27.7, 26.6, 26.3, 24.1, 23.3, 23.2, 18.8, 14.5, 14.4, –5.0.

**HRMS** *m/z* (ESI): calcd. for $C_{38}H_{80}NO_4Si$ $[M+H]^+$: 642.5851; found: 642.5850.

The isolated 6-((4-((tert-butyldimethylsilyl)oxy)butyl)(6-hydroxyhexyl)amino)hexyl (*R*)-2-hexyldecanoate was used for the next Steglich esterification sequence with (*S*)-2-hexyldecanoic acid and the synthesis was completed as mentioned above for (*R*,*R*)-ALC315. 6-((4-((tert-butyldimethylsilyl)oxy)butyl)(6-(((*R*)-2-hexyldecanoyl)oxy)hexyl)amino)hexyl (*S*)-2-hexyldecanoate was obtained as an oil (1.1 g, 37% yield over three steps).

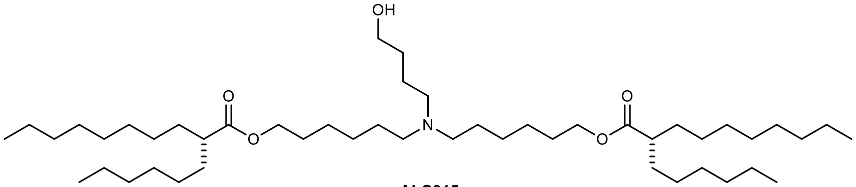

***meso*-ALC315**

**$^1$H NMR** (501 MHz, $CD_2Cl_2$-$d_2$) δ 4.03 (t, *J* = 6.6 Hz, 4H), 3.51–3.44 (m, 2H), 2.45–2.38 (m, 6H), 2.29 (tt, *J* = 8.8, 5.3 Hz, 2H), 1.65–1.22 (m, 69H), 0.88 (t, *J* = 6.9 Hz, 12H).

**$^{13}$C NMR** (126 MHz, $CD_2Cl_2$-$d_2$) δ 176.9, 64.5, 63.1, 55.2, 54.2, 46.4, 33.4, 33.1, 32.5, 32.3, 30.2, 30.0, 29.9, 29.8, 29.3, 28.0, 28.0, 27.9, 26.8, 26.6, 26.5, 23.3, 23.2, 14.5, 14.4.

**HRMS** *m/z* (ESI): calcd. for $C_{48}H_{96}NO_5$ $[M+H]^+$: 766.7283; found: 766.7285.

calculated dr = >20:1.

## 8. Materials and Methods:

### 8a. Construction of ALC315 LNPs:

EGFP was cloned into the pME18S vector containing the T7 promoter, 5’ and 3’ UTRs and a poly(A) tail. EGFP mRNA was synthesized by in vitro transcription (IVT) from the linearized EGFP DNA template using the RiboMAX Large Scale RNA Production Systems (Promega). The EGFP mRNA was capped with CleanCap AG 3'OMe (Trilink), and the UTPs were fully substituted with N1-Methylpseudouridine-5'-Triphosphate (Trilink). The EGFP mRNA was purified using the RNeasy Mini Kit (Qiagen).

The purified EGFP mRNA was dissolved in 50mM sodium acetate buffer (pH 4.0). Lipids, including ALC315 (*S*,*S*), (*R*,*S*), (*R*,*R*), respectively), DSPC, cholesterol, and ALC-0159, were dissolved in ethanol at a molar ratio of 47.5 : 10.0 : 40.7 : 1.8. The mRNA and lipids were mixed in a 3:1 ratio using the NanoAssemblr Ignite system (Cytiva, Canada). The mixture was then dialyzed with 20mM Tris-HCl buffer containing 8% sucrose (pH 7.4) using a Slide-A-Lyzer Dialysis Cassette with 10k MWCO (ThermoFisher Scientific).

### 8b. Cell culture:

The human embryonal kidney cell line HEK293T (ATCC, CRL-1573, ICLAC Accession No. CVCL_3960); human glioblastoma cell lines KMG4 (ICLAC Accession No. CVCL_JZ69) (*1*), U87-MG (ATCC HTB-14), U138-MG (ATCC, HTB16, ICLAC Accession No. CVCL_0020), U343 (Cosmo Bio, Japan, ICLAC Accession No. CVCL_S471); human lung cancer cell line A549 (ATCC, CRM-CCL-185, ICLAC Accession No. CVCL_0023); human bladder cancer cell lines J82 (ATCC, HTB-1, ICLAC Accession No. CVCL_0359); and human cervical cancer cell line HeLa (Riken Cell Bank, RCB0007, ICLAC Accession No. CVCL_0030);human synovial sarcoma cell line SYO-1 (ICLAC Accession No. CVCL_7146) (*2*) were cultured in Dulbecco’s modified Eagle’s medium (DMEM) containing 10% foetal bovine serum (FBS). The human synovial sarcoma cell line Fuji (ICLAC Accession No. CVCL_D880) (*3*); human liver cancer cell lines HepG2 (ATCC, HB-8065, ) and Huh7 (JCRB0403), human colon cancer cell line HCA7 (ICLAC Accession No. CVCL_D0289) ; human T cell leukemia cell line Jurkat (ATCC, TIB-152,) were cultured in Roswell Park Memorial Institute (RPMI) 1640 medium with 10% FBS on the collagen dish.

### 8c. Transfection of GFP LNP:

HEK293T cells were seeded in 24-wel plate with $2.5\times10^5$ cells/0.5 mL/well and after 24 h, 5 µL of GFP-LNPs including ALC315 stereoisomers as (*S*,*S*), (*R*,*S*), and (*R*,*R*) were added to the media and 16 h later, intensities of GFP were measured by FACS. Cells were incubated with propidium iodide and then measured the cell viabilities[1].

## 8d. Phase contrast and fluorescent microscopical analysis of cells:

Photographs of HEK293T cells transfected using LNPs with GFP vectors were excited by 482/35 nm filter and observed by fluorescent microscope EVIDENT-IX73 (EVIDENT, Tokyo, Japan).

## 8e. Immunoblotting:

Cells were lysed with lysis buffer [10 mM Tris-HCl (pH 7.4), 5 mM EDTA, 150 mM NaCl, 10% glycerol, 1% Triton X-100, 1% sodium deoxycholate, 0.1% SDS, 50 mM NaF, 1 mM phenylmethylsulfonyl fluoride (PMSF), and 1 mM sodium orthovanadate ($Na_3VO_4$)] and a protease inhibitor mixture [Complete (EDTA-free) protease inhibitor (Roche, Basel, Switzerland)] for 20 min on ice and centrifuged at 15,000 rpm for 15 min at 4 °C. Supernatants were subjected to SDS-PAGE, and separated proteins were transferred to a polyvinylidene difluoride membrane (Millipore, Billerica, MA). The membranes were incubated with primary antibodies at 4 °C overnight, followed by treatment with horseradish peroxidase-labelled secondary antibodies. The signals were developed using ECLTM Western Blotting Detection Reagents (GE Healthcare, Little Chalfont, UK) and visualized with a LAS 4000 mini (GE Healthcare). Antibodies used in this experiment are as follows: anti-GFP antibody (made by ourselves) and anti-actin antibody (clone C4, MAB1501, Millipore).

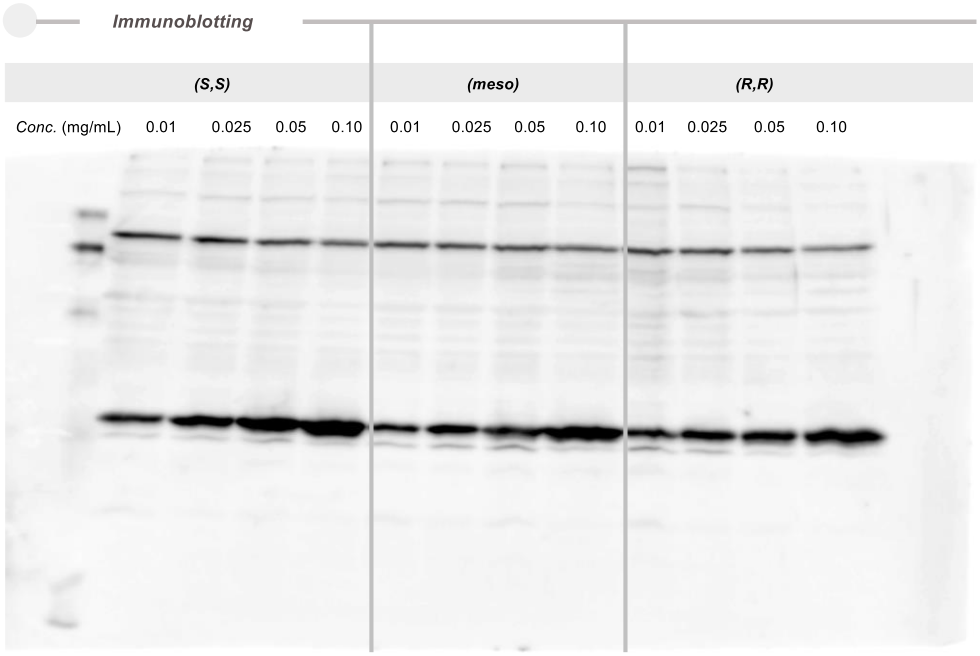


**SI Fig. 2: Transfection of GFP expression plasmid into human embryonic kidney 293T cells with LNPs from stereochemically pure ALC315 isomers.** Immunoblotting of GFP transfected with three isomers of ALC at the indicated concentrations. Arrow indicates GFP as 27 kDa.

## 8f. Cell viability analysis:

The cell viability was evaluated by CCK-8 assay kit (FUJIFILM Wako Pure Chemical Co., Osaka, Japan). Cells were seeded in 96 well plate at the density of $1{\times}10^4$ cells/well. After 24 h of incubation, LNPs containing ALC315 stereoisomers as (*S*,*S*), (*R*,*S*), and (*R*,*R*) at the concentration of 0.05 or 0.1 mg/mL were added to the medium. After additional 48 h, CCK-8 assay was performed and absorbance at 450 nm was measured[2,3].

| | Cell lines | Cancer origin |
|---|---|---|
| 1 | KMG4 | Glioblastoma |
| 2 | U87 | Glioblastoma |
| 3 | U138 | Glioblastoma |
| 4 | A549 | Lung cancer |
| 5 | J82 | Bladder caner |
| 6 | HeLa | Cervical cancer |
| 7 | SYO-1 | Synovial sarcoma |
| 8 | Fuji | Synovial sarcoma |
| 9 | HepG2 | Liver cancer |
| 10 | Huh7 | Liver cancer |
| 11 | HCA7 | Colon cancer |
| 12 | Jurkat | T cell lymphoma |

**SI. Table 1: Human Cell lines treated with ALC isomers.** Twelve human cancer cell lines (summarized in **Table 1**)

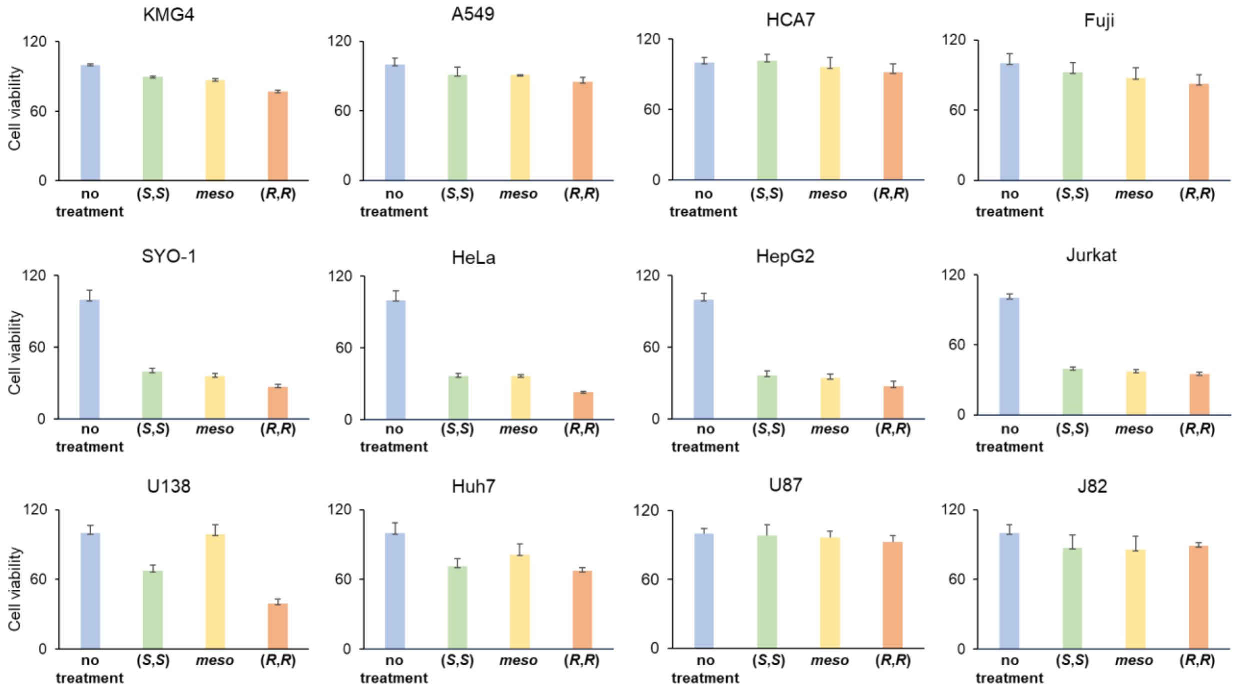


**SI. Fig. 3: Cell viabilities of various human cell lines treated with ALC-isomers.** Twelve human cancer cell lines (summarized in **Table 1**) were incubated with three stereoisomers (*S*,*S*)-ALC315 (green bar), (*S*,*R*)-ALC315 (blue bar), and (*R*,*R*)-ALC315 (red bar) for 48 h and cell viabilities were measured by CCK-8 assay.

## 8g. RNA extraction and gene expression analysis:

Total RNA from cells was extracted using an RNeasy Mini Kit (Qiagen, Valencia, CA) according to the manufacturer's protocols and reverse transcribed into cDNA using the SuperScript® VILO™ cDNA Synthesis Kit (Thermo Fisher Scientific, Waltham, MA). Real-time PCR analysis was performed using the StepOnePlus™ Real-Time PCR System (Applied Biosystems, Waltham, MA) using Power SYBR® Green Master Mix (Thermo Fisher Scientific). Relative expression levels of RNA were normalized to *glyceraldehyde-3-phosphate dehydrogenase* (*GAPDH*). The primers used in this study were as follows: *IL-6*: forward (5'-/////////-3'), reverse (5'-/////////-3'); *TNF-$\alpha$*: forward (5'-/////////-3'), reverse (5'-/////////-3'); *IFN-$\gamma$-R1*: forward (5'-/////////-3'), reverse (5'-/////////-3'); and GAPDH: forward (5'-/////////-3'), reverse (5'-/////////-3').

# 9. NMR traces:

$^{1}H$ and $^{13}C$ NMR traces of **5**

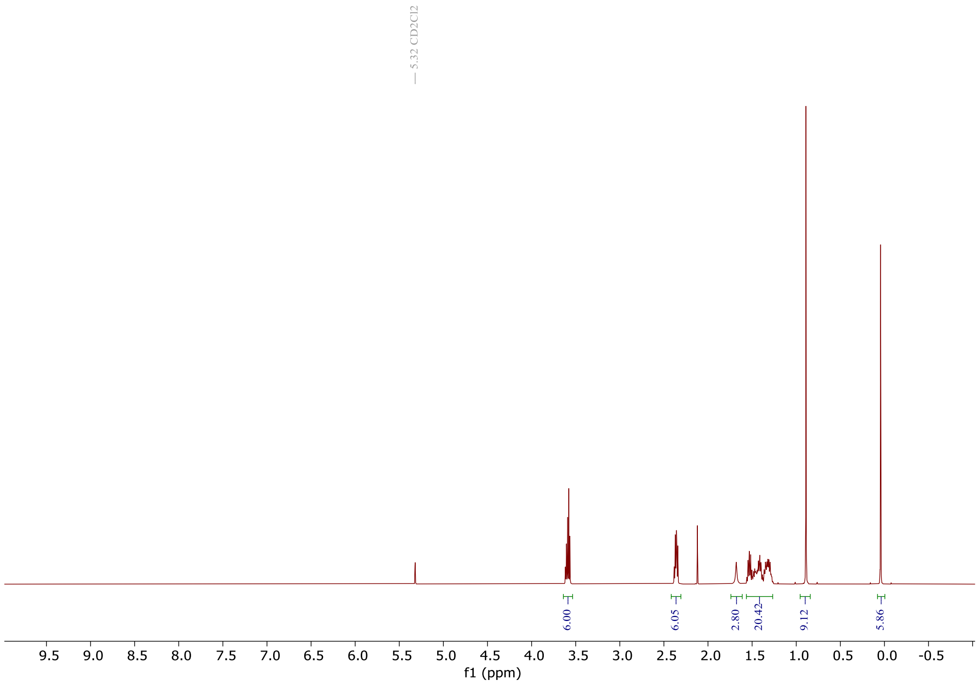


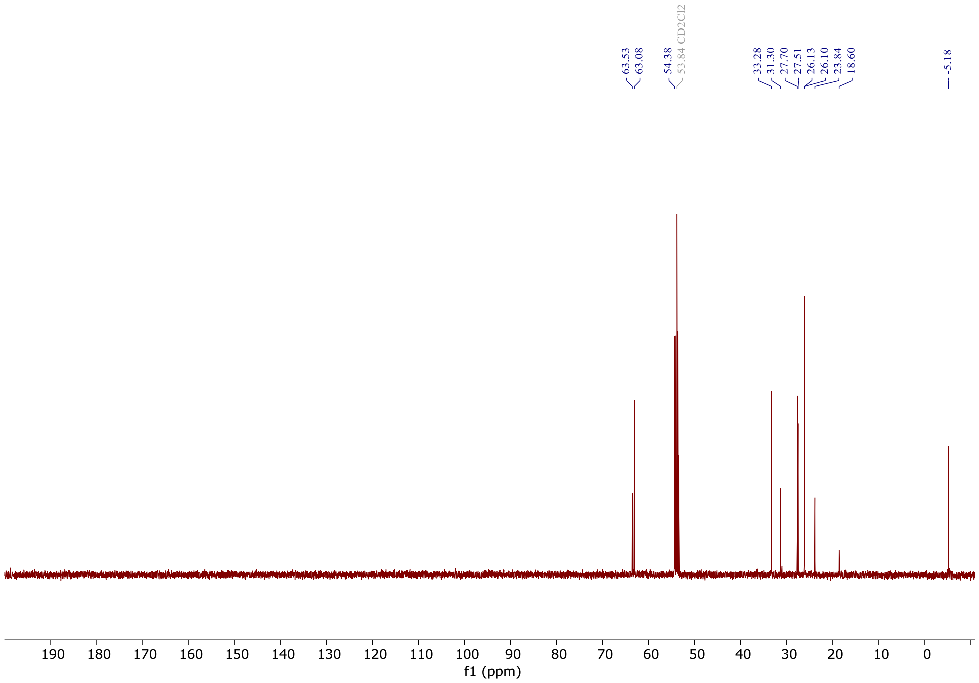

[1]H traces of **7**

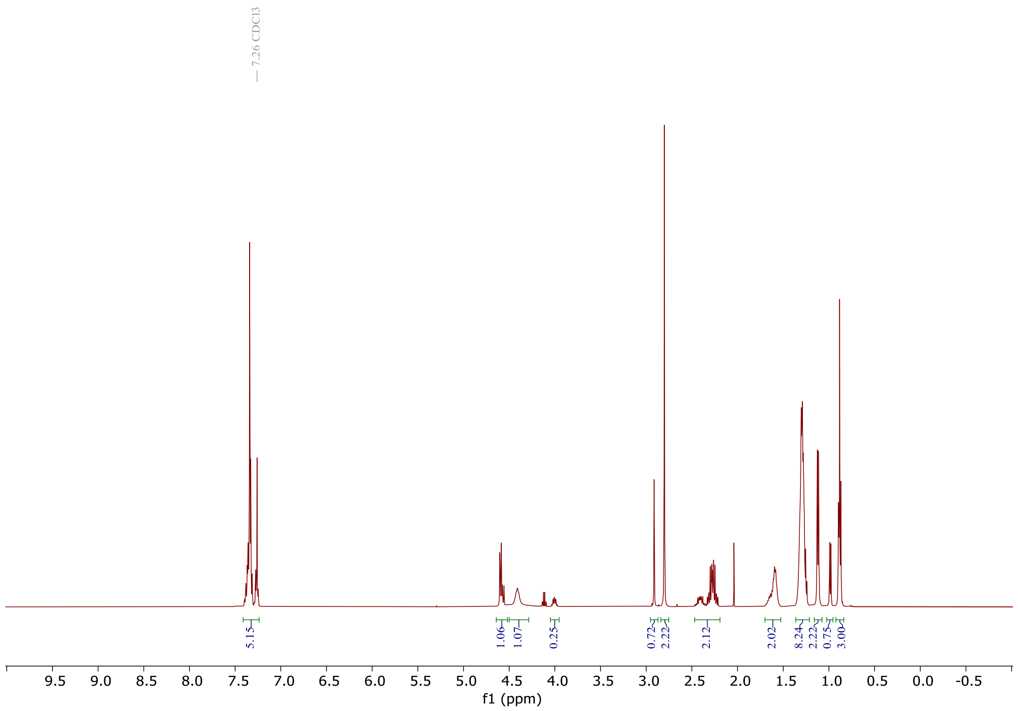


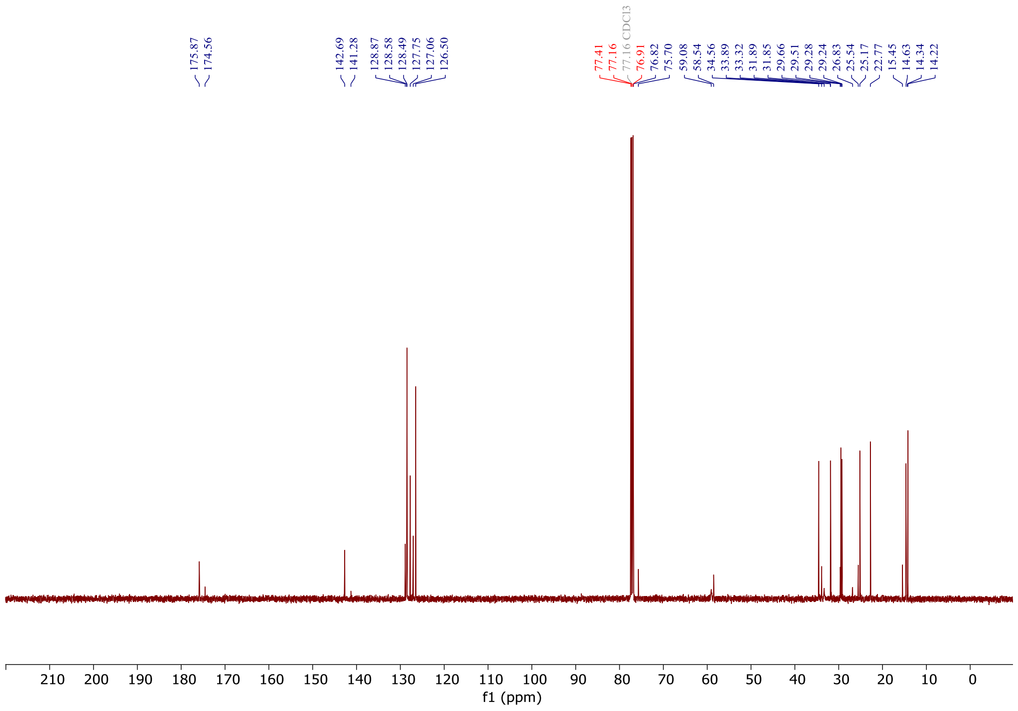

$^{1}H$ and $^{13}C$ NMR traces of **8**

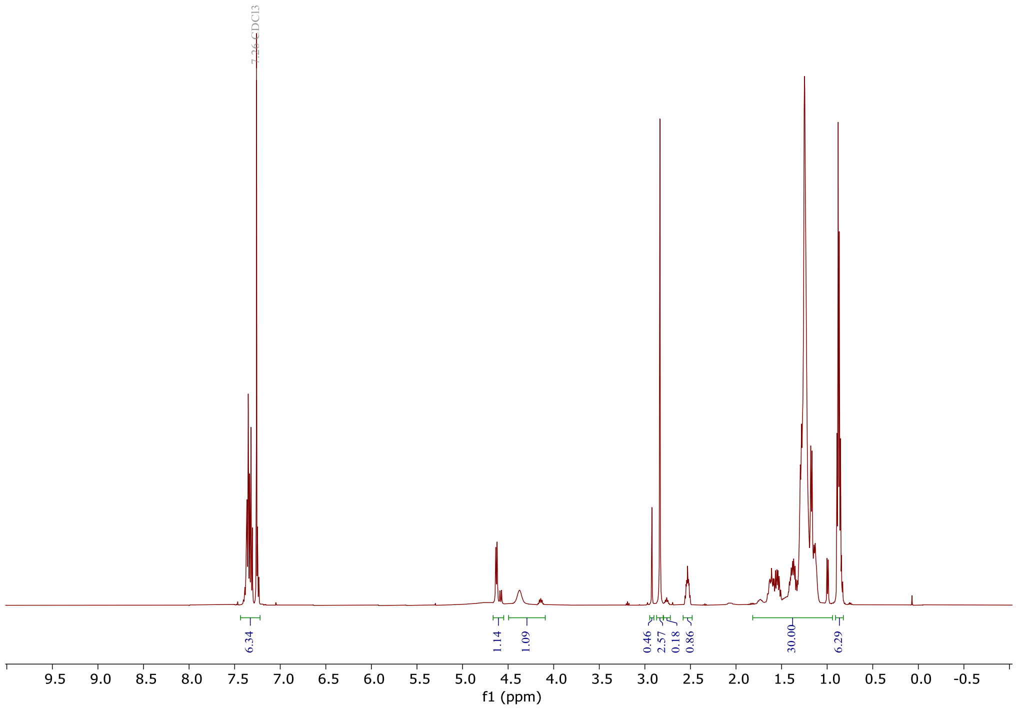

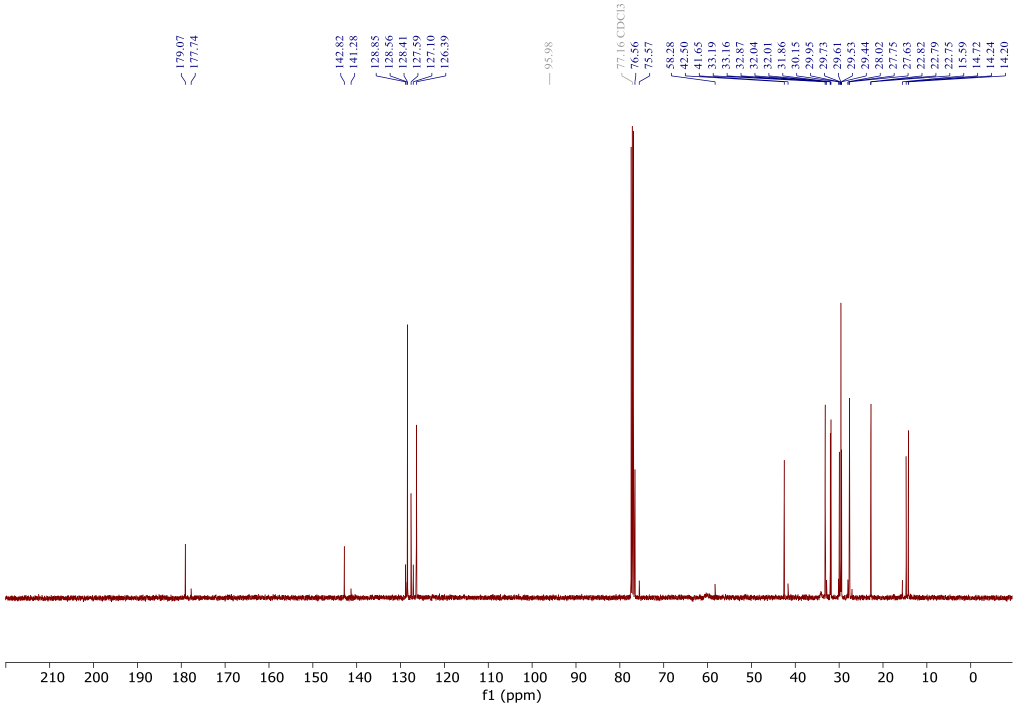

$^{1}H$ and $^{13}C$ NMR traces of ***(R)*-1**

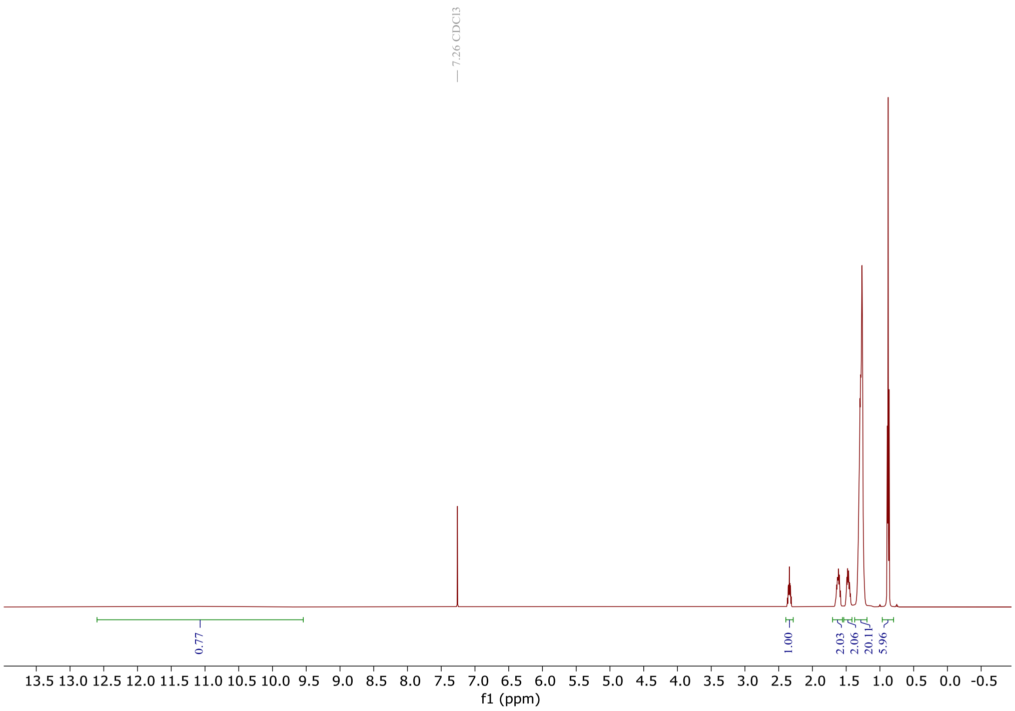


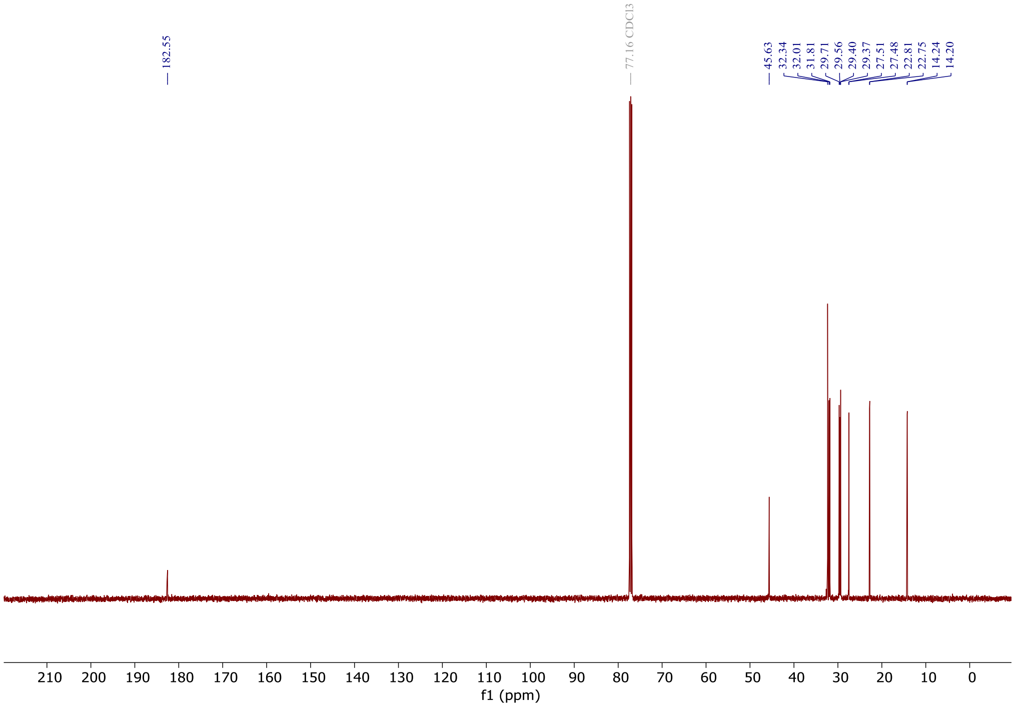

[1]H traces of **9**

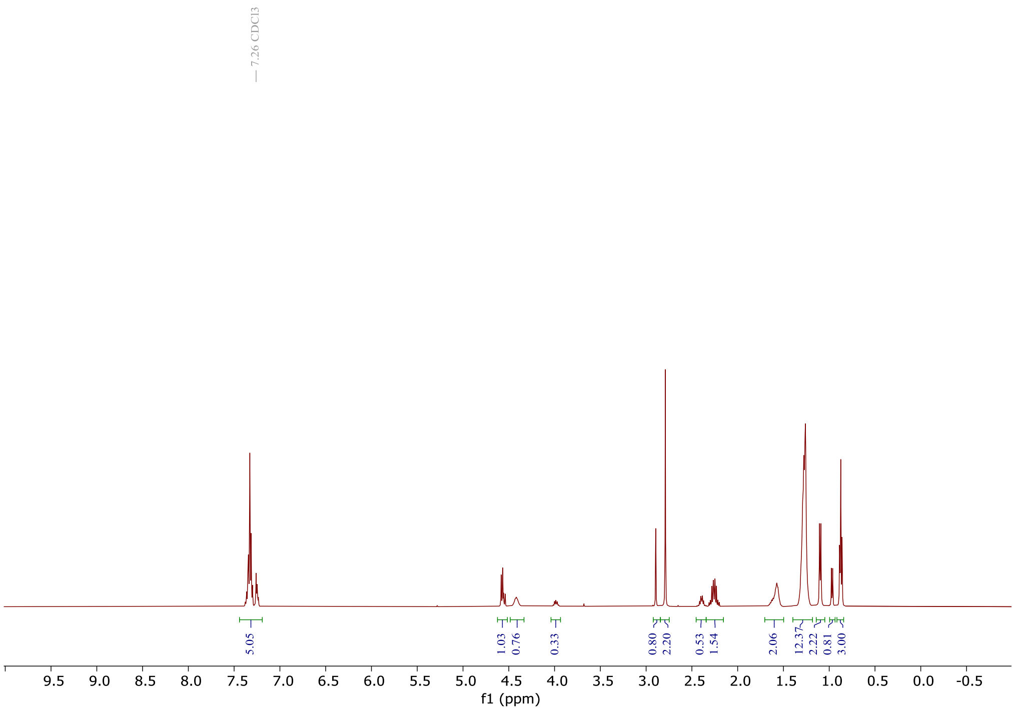


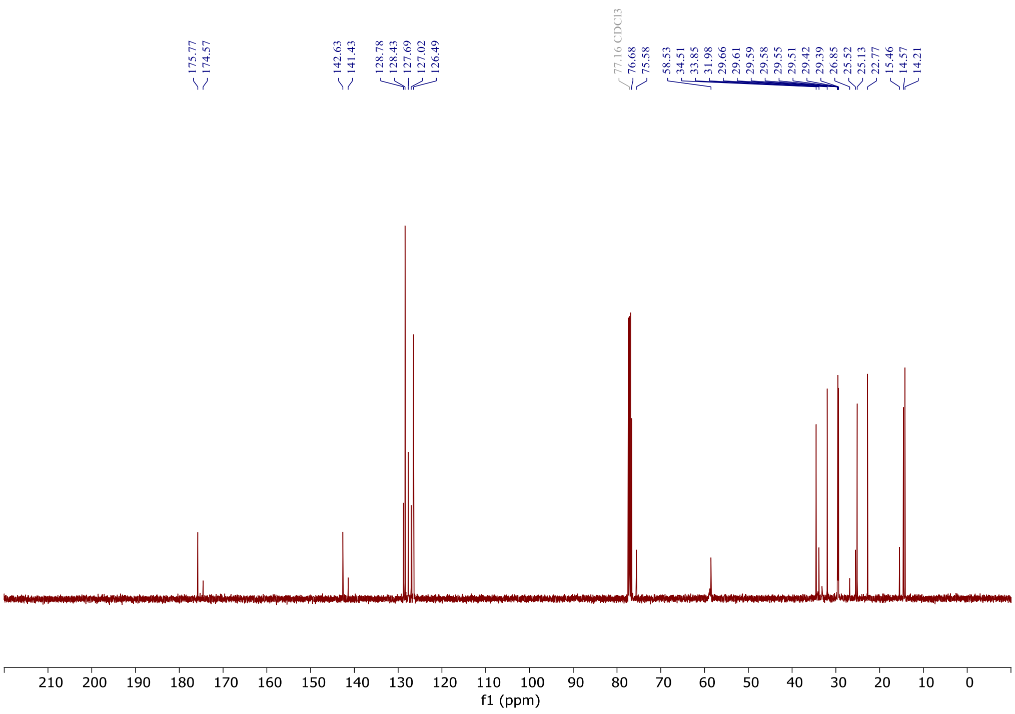

$^{1}$H and $^{13}$C NMR traces of **10**

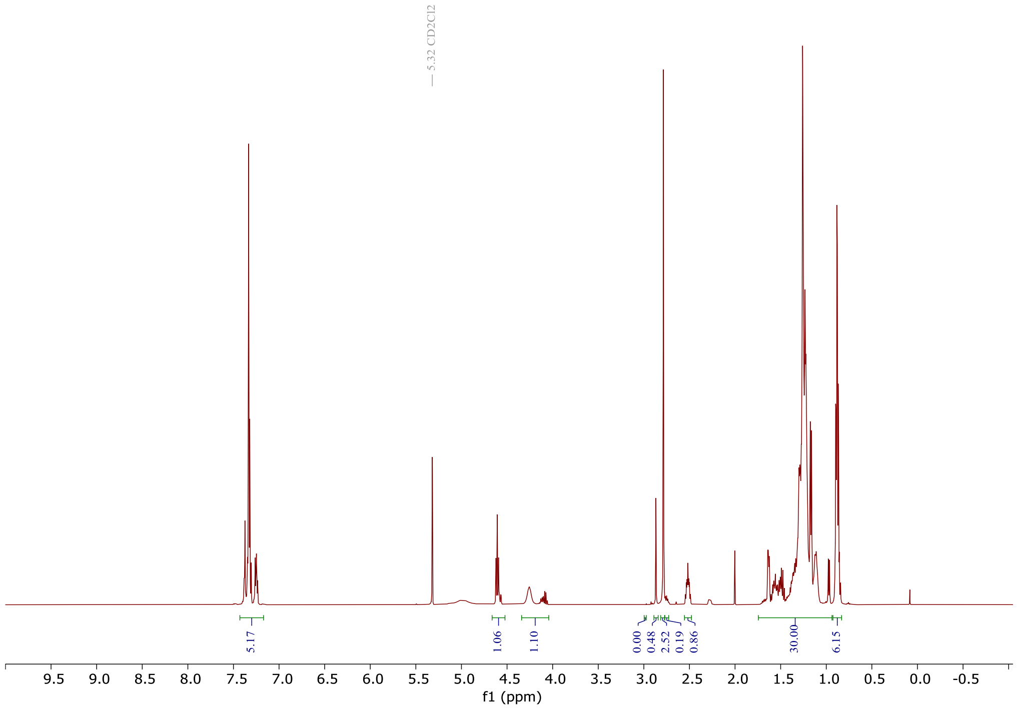


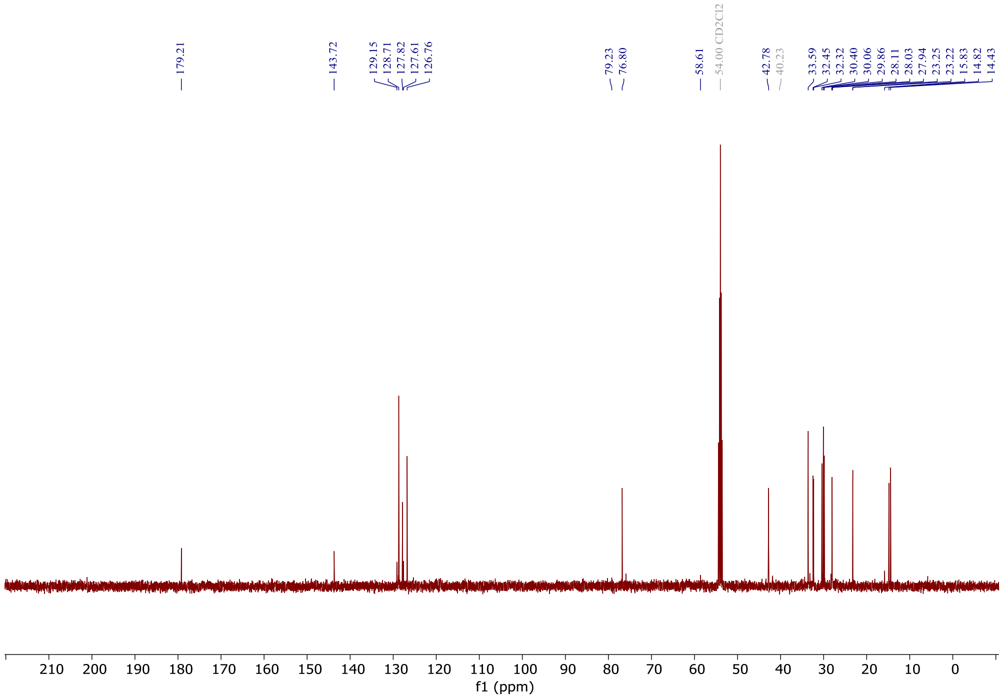

$^{1}$H and $^{13}$C NMR traces of **(*S*)-1**

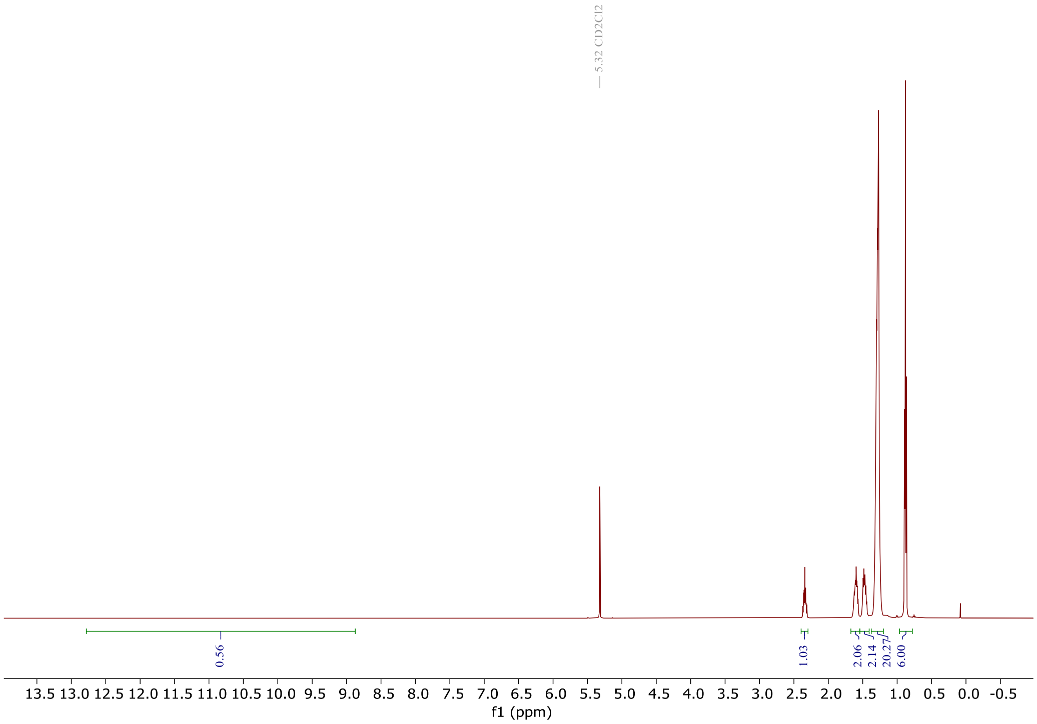


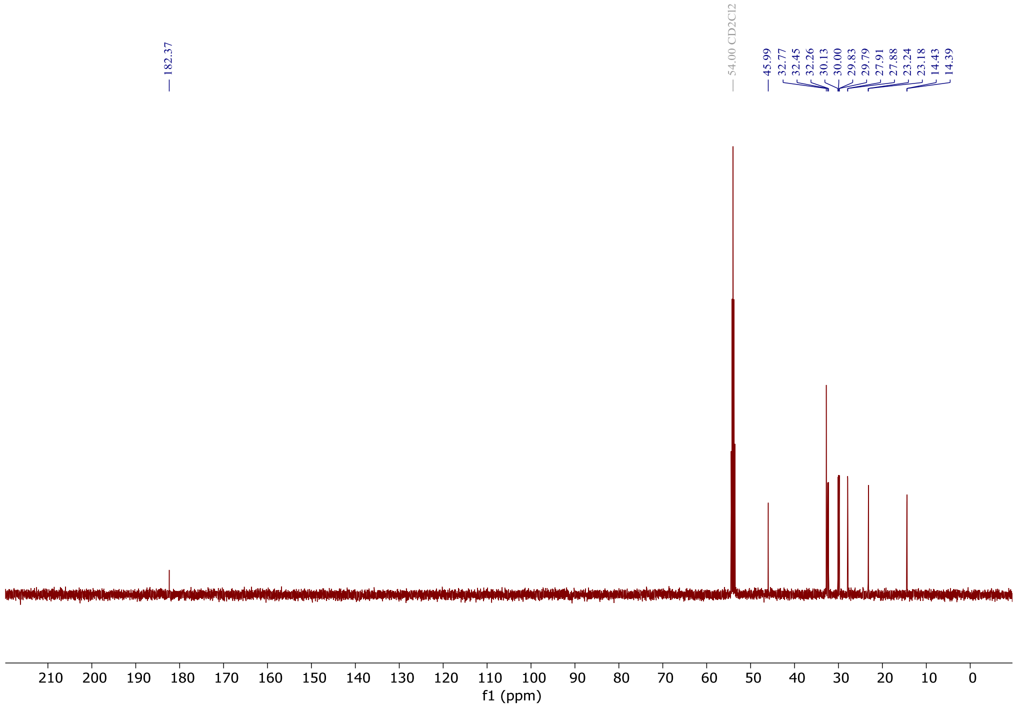

$^{1}$H and $^{13}$C NMR traces of **(*R*,*R*)-ALC315**

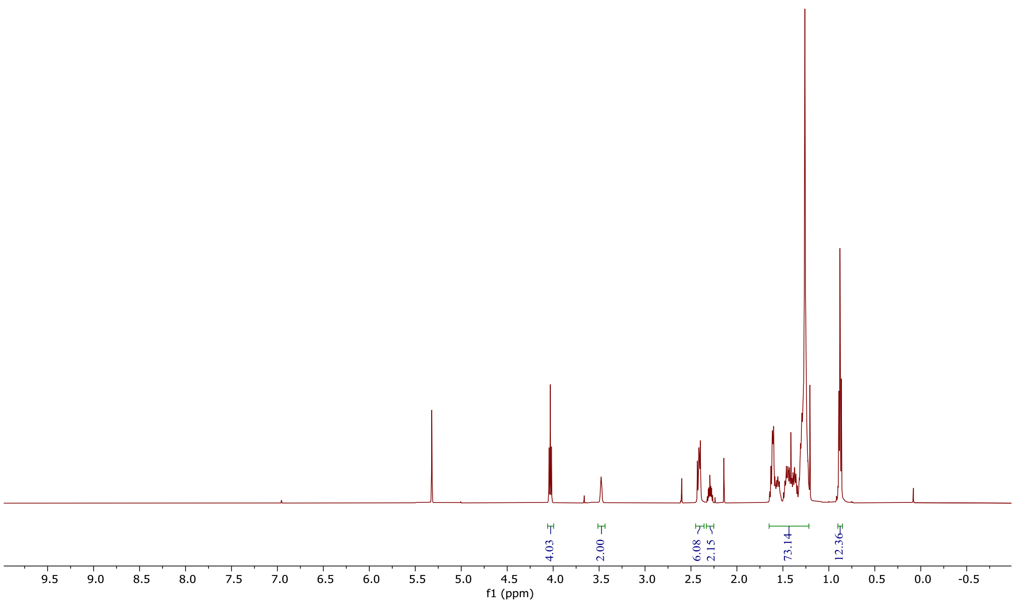


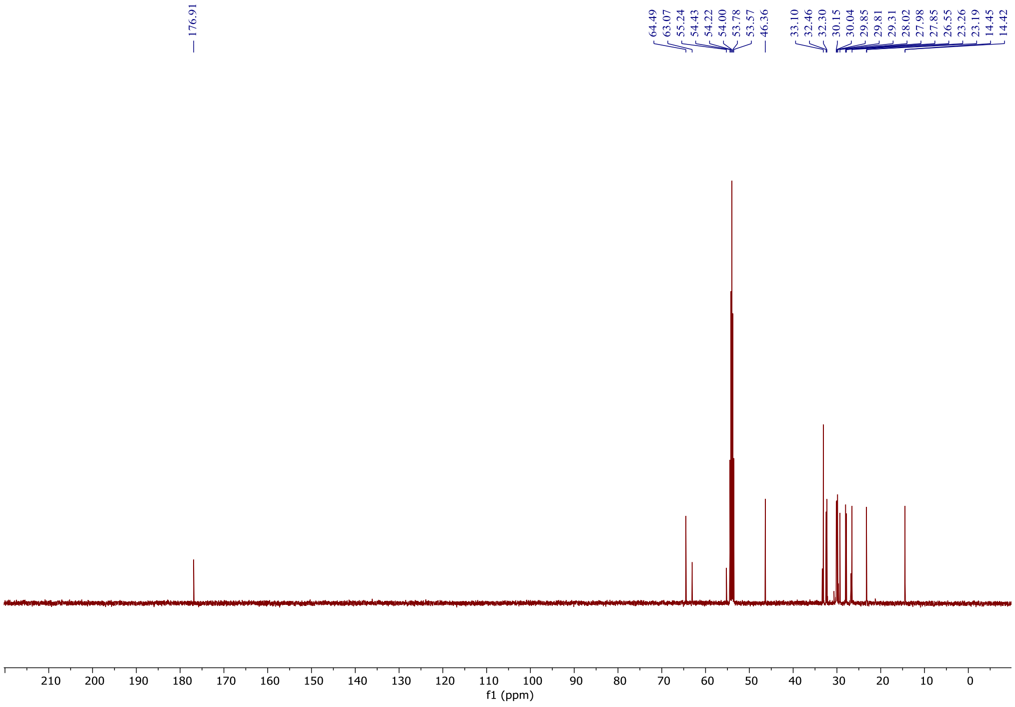

$^{1}$H and $^{13}$C NMR traces of ***(S,S)*-ALC315**

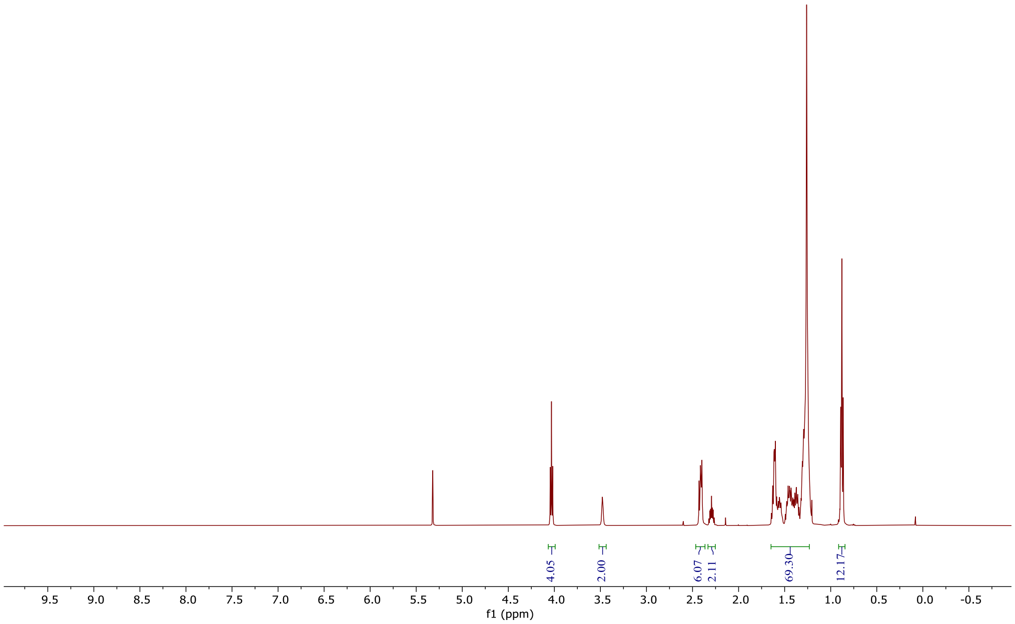


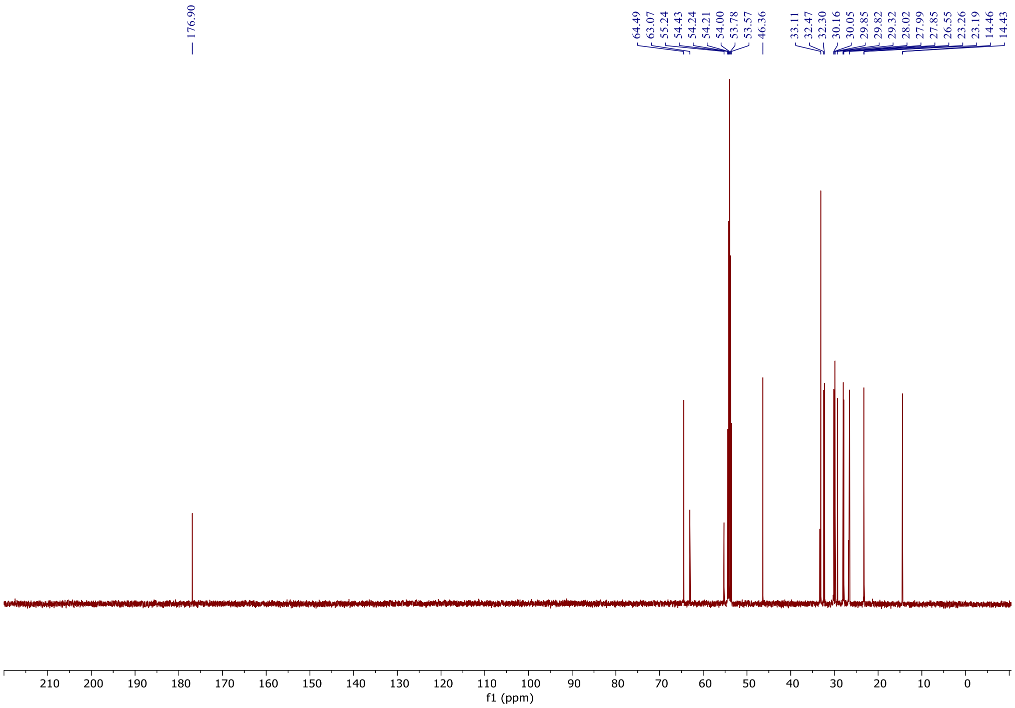

$^{1}$H and $^{13}$C NMR traces of **6**

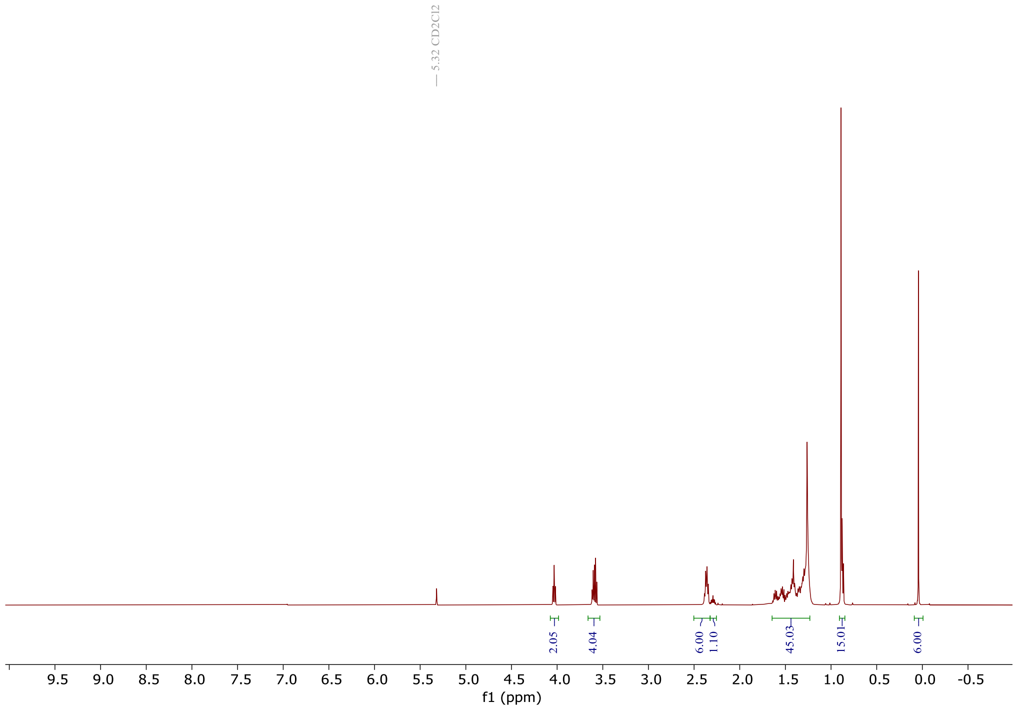


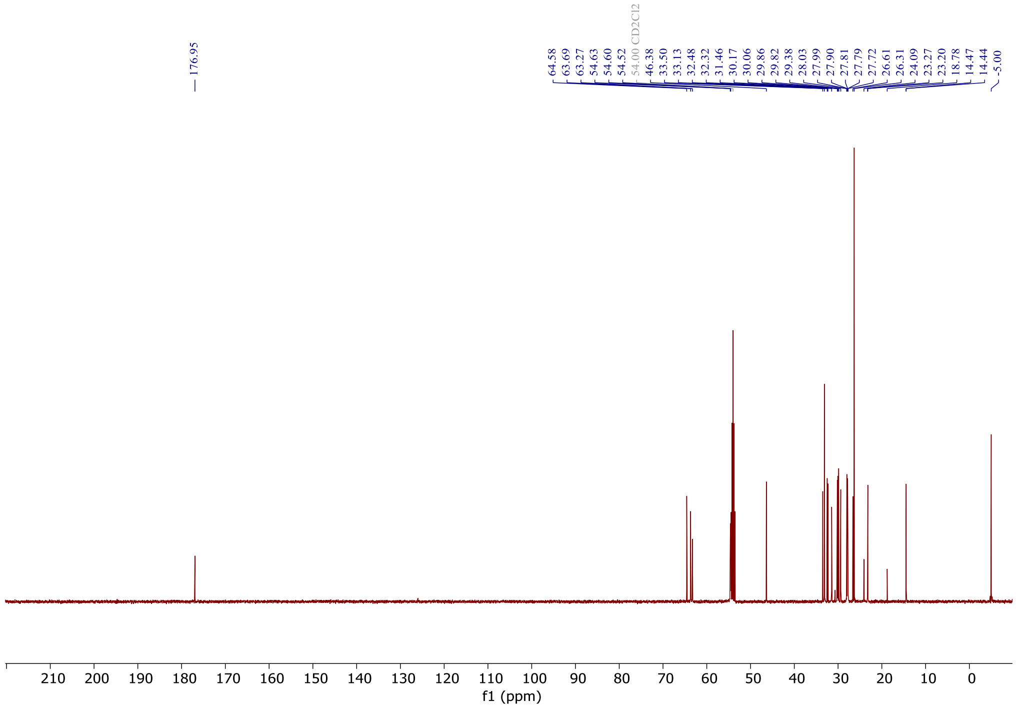

$^{1}$H and $^{13}$C NMR traces of ***meso*-ALC315**

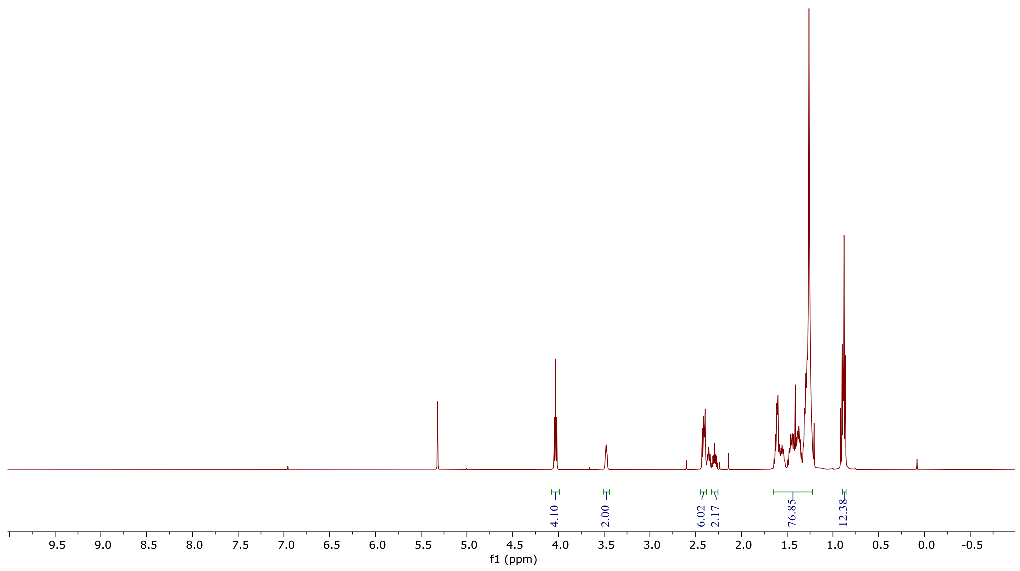


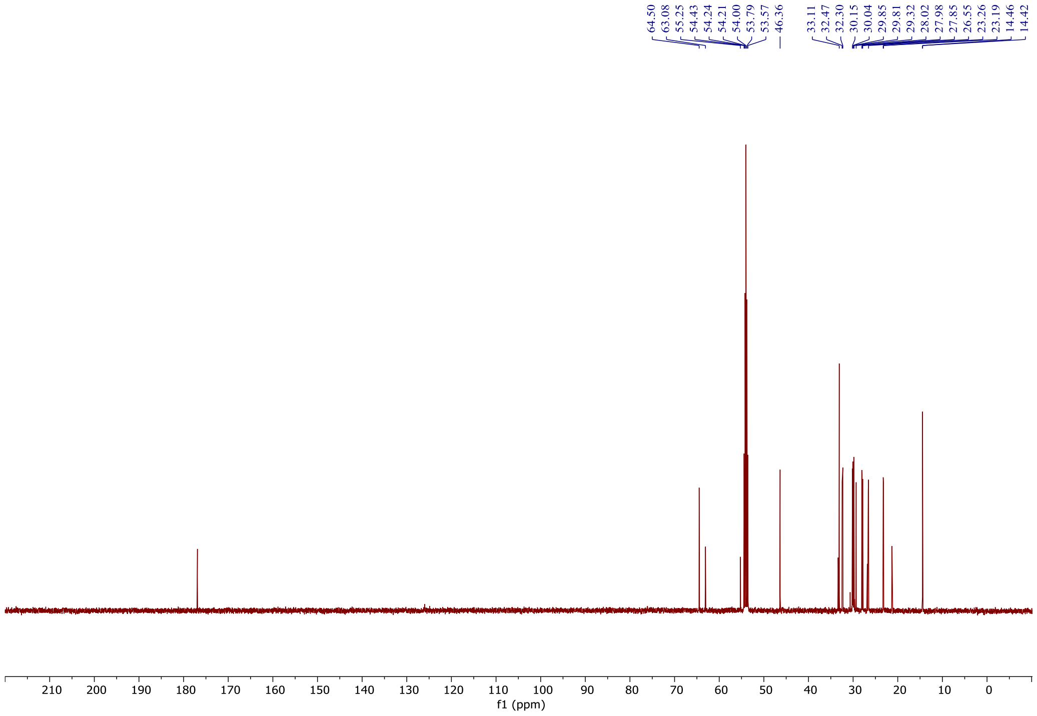

## 10. HPLC traces:

HPLC trace of *rac*-**1** acid derivative (amide) with (*R*)-1-Phenylethylamine

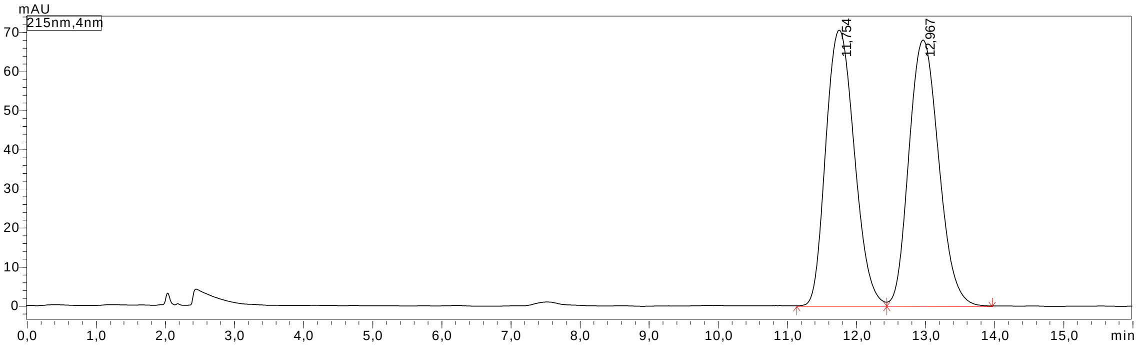


HPLC trace of (*R*)-**1** acid derivative (amide) with (*R*)-1-Phenylethylamine

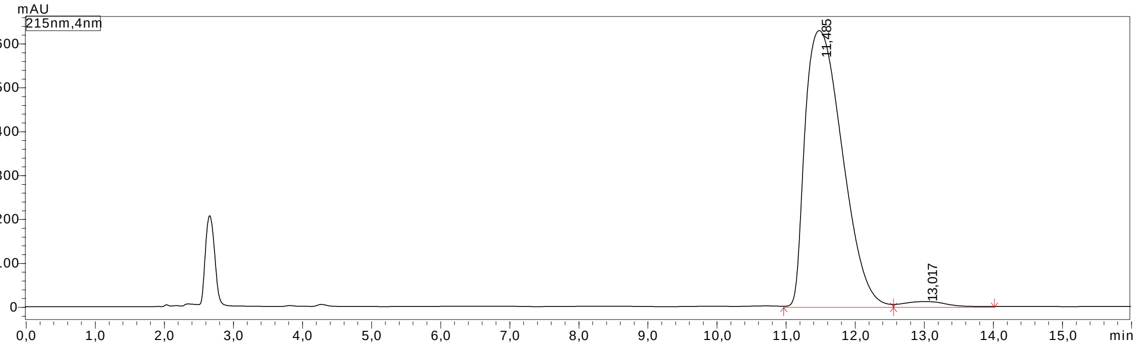


HPLC trace of (*S*)-**1** acid derivative (amide) with (*R*)-1-Phenylethylamine

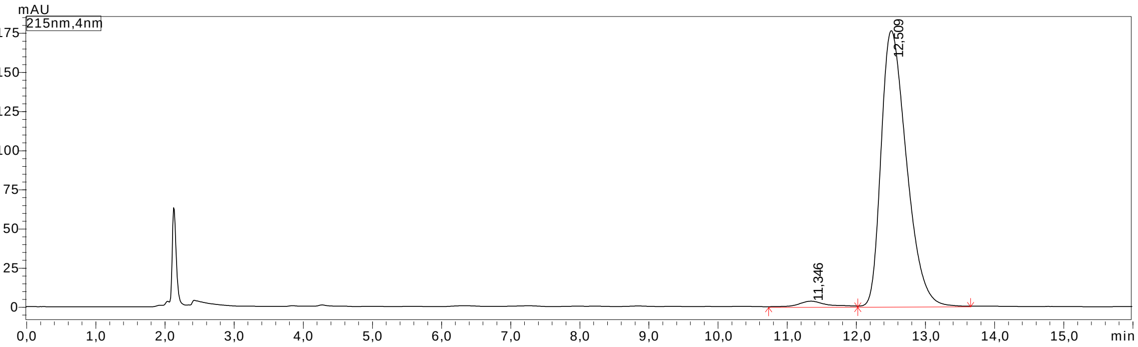


HPLC trace of *rac*-**1** acid

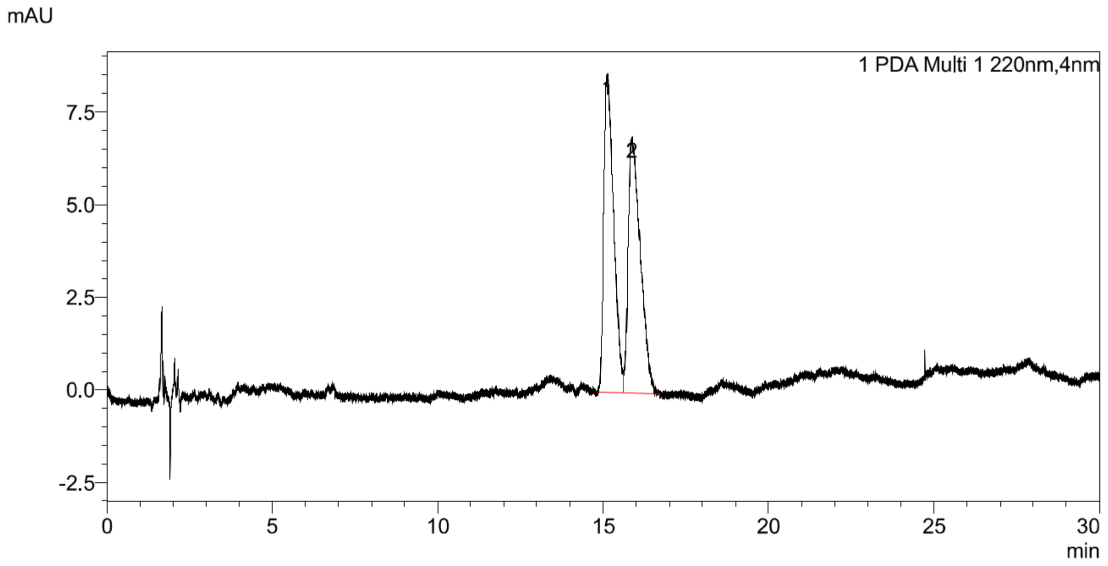
mAU
1 PDA Multi 1 220nm,4nm
7.5
5.0
2.5
0.0
-2.5
1
2
0
5
10
15
20
25
30
min

## 11. Supplementary References:


1. Momozaki, N.; Oh-Uchida, M.; Tabuchi, K.; Ikezaki, K.; Hori, K. Suppression of Anchorage-Independent Growth of Human Glioblastoma Cell by Major Histocompatibility Complex Class I Gene-Transfection. *J. Neurosurg.* **1992,** *76,* 845–849.

2. Kawai, A.; Naito, N.; Yoshida, A.; Morimoto, Y.; Ouchida, M.; Shimizu, K.; Beppu, Y. Establishment and Characterization of a Biphasic Synovial Sarcoma Cell Line, SYO-1. *Cancer Lett.* **2004,** *204*, 105–113.

3. Nojima, T.; Wang, Y. S.; Abe, S.; Matsuno, T.; Yamawaki, S.; Nagashima, K. Morphological and Cytogenetic Studies of a Human Synovial Sarcoma Xenotransplanted into Nude Mice. *Acta Pathol. Jpn.* **1990,** *40,* 486–493.
